# Synthesis, characterization, biological potency, and molecular docking of Co^2+^, Ni^2+^ and Cu^2+^ complexes of a benzoyl isothiocyanate based ligand

**DOI:** 10.1038/s41598-024-58108-5

**Published:** 2024-05-01

**Authors:** Eida S. Al-Farraj, Adel M. Younis, Gaber M. I. Abu El-Reash

**Affiliations:** 1 Department of Chemistry, College of Science, Imam Mohammad Ibn Saud Islamic University (IMSIU), 11623 Riyadh, Saudi Arabia; 2 Department of Chemistry, Faculty of Science, Mansoura University, Mansoura, Egypt

**Keywords:** Quantum chemical computations, Thermodynamic and kinetic stability, Absorption and emission spectroscopy, Biological activity, Molecular docking, Bioinorganic chemistry, Coordination chemistry, Inorganic chemistry

## Abstract

The primary objective of the present study was to produce metal complexes of H_4_DAP ligand (N,N'-((pyridine-2,6-diylbis(azanediyl))bis(carbonothioyl))dibenzamide) derived from 2,6-diaminopyridine and benzoyl isothiocyanate with either ML or M_2_L stoichiometry. There are three distinct coordination complexes obtained with the formulas [Co(H_2_DAP)]·H_2_O, [Ni_2_(H_2_DAP)Cl_2_(H_2_O)_2_]·H_2_O, and [Cu(H_4_DAP)Cl_2_]·3H_2_O. The confirmation of the structures of all derivatives was achieved through the utilization of several analytical techniques, including FT-IR, UV–Vis, NMR, GC–MS, PXRD, SEM, TEM analysis, and QM calculations. Aiming to analyze various noncovalent interactions, topological methods such as QTAIM, NCI, ELF, and LOL were performed. Furthermore, the capacity of metal–ligand binding was examined by fluorescence emission spectroscopy. An in vitro investigation showed that the viability of MDA-MB-231 and HepG-2 cells was lower when exposed to the manufactured Cu^2+^ complex, in comparison to the normal cis-platin medication. The compounds were further evaluated for their in vitro antibacterial activity. The Ni^2+^ complex has shown promising activity against all tested pathogens, comparable to the reference drugs Gentamycin and Ketoconazole. Furthermore, a computational docking investigation was conducted to further examine the orientation, interaction, and conformation of the recently created compounds on the active site of the Bcl-2 protein.

## Introduction

Heterocycles are highly active parts of cancer medications, some of which are linked to metallic complexes. For instance, nitrogen-donor chelating ligands have been frequently exploited in the architectural design of metal complexes with biological purposes, comprising cytotoxic actions^1–3^. In this case, an array of 5-iodouracil complexes with Co^2+^ and Cu^2+^ ions were produced, some of which were potent against Sarcoma-180 and L929 cell tumors^4^. Numerous research studies demonstrate that cobalt, nickel, and copper compounds have a better biological profile as anticonvulsive, anti-inflammatory, antibacterial, and antifungal agents^5^. Numerous clinical studies have emphasized that complexes play a fascinating role in the creation of a range of anti-tumor medicines, and the variety of them additionally relies on the ligand coupled to the metal^6^. An important characteristic of copper derivatives is their usage in producing anticancer medications, which could be an option in addition to platinum-based therapeutics since copper compounds aren't as toxic and their mode of action is relatively simpler than other therapies for cancer^7^. It was observed that copper complexes, including pyridyl ligands, suppress breast cancer cells^8^.

Isothiocyanates (ITCs) represent the subsequent intermediates of Cruciferae with antibacterial properties^9^. Nowadays, over 120 varieties of ITCs have been identified in vegetation, and benzyl isothiocyanate (BITC) represents one of the primary bioactive parts of ITCs^10^, which may be isolated from harmless food sources with antibacterial capabilities. BITC has been observed to prevent the spread of pathogens^11^, such as molds, bacteria, and parasites, and it has garnered widespread interest owing to its efficacy as an alternative antibacterial component. Some researchers have demonstrated that BITC can impact the composition and operation of the cell membrane and the protein expression patterns of microorganisms^12^.

Several substances having a pyridine backbone have been reported to be physiologically active^13,14^ and therapeutically useful in the field of medicinal chemistry and to demonstrate varied biological capabilities, including antimicrobial and cancer fighting abilities^13–17^.

Due to their substantial biological activity, the combination of ICTs with pyridine components could contribute to the creation of a category of biologically active molecules, making it very desirable.

To achieve this goal, we produced an analog of the N, O, and S-chelating isothiocyanate-pyridine ligand of the H_4_DAP junction to examine its in vitro antibacterial, cytotoxic, and antioxidant properties, with a focus on their Co^2+^, Ni^2+^, and Cu^2+^ complexes. This led us to formulate the N,N'-((pyridine-2,6-diylbis(azanediyl))bis(carbonothioyl))dibenzamide ligand and its [Co(H_2_DAP)]·H_2_O, [Ni_2_(H_2_DAP)Cl_2_(H_2_O)_2_]·H_2_O, and [Cu(H_4_DAP)Cl_2_].3H_2_O metal complexes.

## Experimental

### Materials and instruments

All utilized starting ingredients—benzoyl isothiocyanate, 2,6-diaminopyridine, metallic salts, and solvents—were bought from viable vendors (Merck or Sigma-Aldrich) and used instantly with no extra purifications. The molecular structures of the isolated substances have been clarified through the subsequent strategies:

**Table:** 

C, H, N, and S content	C, H, N, and S contents were obtained using Thermo-Fisher Scientific Analyzer Model: Flash 2000
Metal and Cl^¯^ contents	Using described approaches in Vogel's Textbook of Quantitative Chemical Analysis^18^
UV–Vis	Unicam UV–Vis spec. in 1 × 10^−4^ M concentration in DMSO solvent
FT-IR spectra	Mattson 5000 (4000–400cm^−1^, KBr discs)
Mass spectra	DI-50 unit-Shimadzu GC–MS-QP5050A
Magnetic moment	Sherwood magnetic balance at 32 °C
^1^H, ^13^C-NMR	JEOL ECA-500 II
P-XRD	Shimadzu XRD 6000 diffractometer (Japan), Cu anode, K_a_: 0.154060 nm, 2θ = 5–80°
TGA	Perkin Elmer TGA 4000, 30–900 °C, N_2_ flow 20 ml/min, Rate of heating 10 °C/min
Morphology studies	SEM, JOEL JSM 6510 lv. TEM, JOEL JEM 2100

### Synthesis of H_4_DAP ligand and its metallic derivatives

#### Synthesis of H_4_DAP ligand

**Figure 1:**
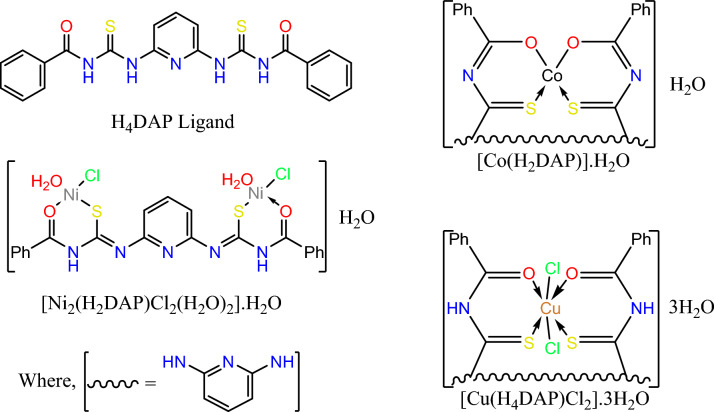
Structures of H_4_DAP ligand and its complexes.

H_4_DAP ligand (Fig. 1) was produced by adding 2 mmol of benzoyl isothiocyanate to 1 mmol of 2,6-diaminopyridine in 20 mL of pure ethanol. A firm precipitate began to be produced within 30 min. The reaction solution was agitated for an extra 2 h to guarantee full production of the ligand. The resulting gray precipitate was filtered out, rinsed with warm ethanol and diethyl ether, and finally dried in vacuum on CaCl_2_ anhydrous. The chemical purity of the ligand was evaluated by TLC. (%Yield = 86), color: grey, m.p. > 300 °C. Elemental analyses: C (found = 57.12%, calc. = 57.91), H (found = 3.63%, calc. = 3.93%), N (found = 16.86, calc. = 16.08%), and S (found = 14.15%, calc. = 14.72%), and m/z = 435.95 (18.53%) matches with (C_21_H_17_N_5_O_2_S_2_, F.W. = 435.52) Figure S1.

#### Synthesis of the metal complexes (Fig. 1)

An ethanol-based solution of metallic salt, CoCl_2_, NiCl_2_.6H_2_O, and CuCl_2_.6H_2_O, was combined with an ethanol solution containing the ligand in a 1:1 molar proportion and refluxed while stirring for 5h. The resulting solid metallic complexes were filtered out, rinsed with heated ethanol and diethyl ether, respectively, and finally dried over CaCl_2_ (anh.).

- *Co*^*2+*^* complex*: %Yield = 81, color: greenish blue, m.p. > 300 °C. Elemental analyses: C (found = 49.92%, calc. = 49.41), H (found = 3.23%, calc. = 3.36%), N (found = 13.91, calc. = 13.72%), S (found = 12.27%, calc. = 12.56%), and Co (found = 11.23%, calc. = 11.55%), and m/z = 510.89 (47.44%) matches with (C_21_H_17_CoN_5_O_3_S_2_, F.W. 510.45), Figure S2.
- *Ni*^*2+*^ *complex*: %Yield = 76, color: green, m.p. > 300 °C. Elemental analyses: C (found = 37.95%, calc. = 37.34), H (found = 2.91%, calc. = 3.13%), N (found = 10.84, calc. = 10.36%), S (found = 9.27%, calc. = 9.49%), and Ni (found = 17.23%, calc. = 17.37%), and m/z = 675.72 (18.80%) matches with (C_21_H_21_Cl_2_N_5_Ni_2_O_5_S_2_, F.W. 675.84), Figure S3.
- *Cu*^*2+*^* complex*: %Yield = 63, color: Brown, m.p. > 300 °C. Elemental analyses: C (found = 40.58%, calc. = 40.42%), H (found = 3.53%, calc. = 3.72%), N (found = 11.27, calc. = 11.22%), S (found = 10.16%, calc. = 10.28%), and Cu (found = 10.58%, calc. = 10.18%), and m/z = 624.35 (26.37%) matches with (C_21_H_23_Cl_2_N_5_CuO_5_S_2_, F.W. 624.01), Figure S4.

### QM calculations

DFT simulations were done to understand the approach of binding and verify the intended geometries of the molecules under inquiry. The DFT computations were carried out utilizing the DMol^3^/BIOVIA-MS platform for the separated metallic complexes via GGA/RPBE functionality and the DNP basis set. DFT parameters were obtained by employing HOMO and LUMO values of energy to represent the chemical reactivity of the ligand and its complexes with metals^19–22^. Topological (QTAIM, LOL, and ELF) and NCI studies were conducted utilizing Multiwfn software^23^.

### Molecular docking

A computational docking study was conducted by the MOE program for Windows^24^ to identify the affinity for binding of the compounds that were isolated to the target site of Bcl2 (pdb code: 2W3L) substrates^25^. The co-crystallized ligand DRO was used as a reference. The co-crystallized ligand DRO and the compounds under investigation were brought into MOE, then underwent 3D protonation and energy minimization, subsequently loaded into an identical database, and stored in the format of an MDB. The target’s structural data was acquired from the Protein Data Bank at satisfactory qualities of 2.10 Å and loaded into MOE; thereafter, the structure creation algorithm of MOE was utilized to rectify all the flaws in the structure of the protein. Hydrogen atoms were introduced to structures in their standard form, and all molecules of solvent were eliminated from the frameworks, which then underwent a minimization of energy. The final optimized structures have been preserved. Triangle matcher and refining approaches were employed for undertaking docking tests. After finishing the docking operations, the resulting poses were evaluated, and the most suitable ones exhibiting the best acceptable rmsd_refine ratios with the identical interaction mode of the native ligand were chosen.

### Biological applications

#### Antimicrobial activity

The antimicrobial activities of the H_4_DAP ligand and its complexes were investigated in laboratory conditions against specific types of fungi (*Aspergillus fumigatus* and *Candida albicans*), gram-positive bacteria (*Staphylococcus aureus* and *Bacillus subtilis*), and gram-negative bacteria (*Proteus vulgaris* and *Escherichia coli*) using established methods^26,27^. Specific steps are in the supplementary data.

#### Cytotoxic activity

The prepared H_4_DAP ligand and its complexes with metals were investigated for in vitro cytotoxicity utilizing the MTT, and crystal violet examinations targeting human HepG-2 (acquired from the ATCC American Type Culture Collection) and MDA-MB-231 (acquired from the VACSERA Tissue Culture Unit) cell lines^28–30^. Specific steps are in the supplementary data.

#### Antioxidant activity

The prepared H_4_DAP ligand and its metallic complexes have been examined for their antioxidant capabilities. The antioxidant activities of the evaluated compounds were assessed using three approaches: DPPH, FRAP, and ABTS scavenging activities. Ascorbic acid was employed as a standard substance for evaluating the antioxidant activity findings^31–33^. The assay protocols can be found in the Supplementary Information section.

## Results and discussions

### Structures elucidation

The structures of the compounds were validated using multiple methodologies (FT-IR, NMR, UV–vis, GC–MS, TGA, and PXRD). The acquired data revealed a successful interaction between the ligand and the metal salts, and matched with the planned formulas of the complexes, and indicated that the ligand interacted with the metal salts in a 1:1 mol ratio, with the exception of the Ni^2+^ complex, which is generated in a 1:2 (L:M) mole ratio.

#### Characterization of the H_4_DAP ligand

**Figure 2:**
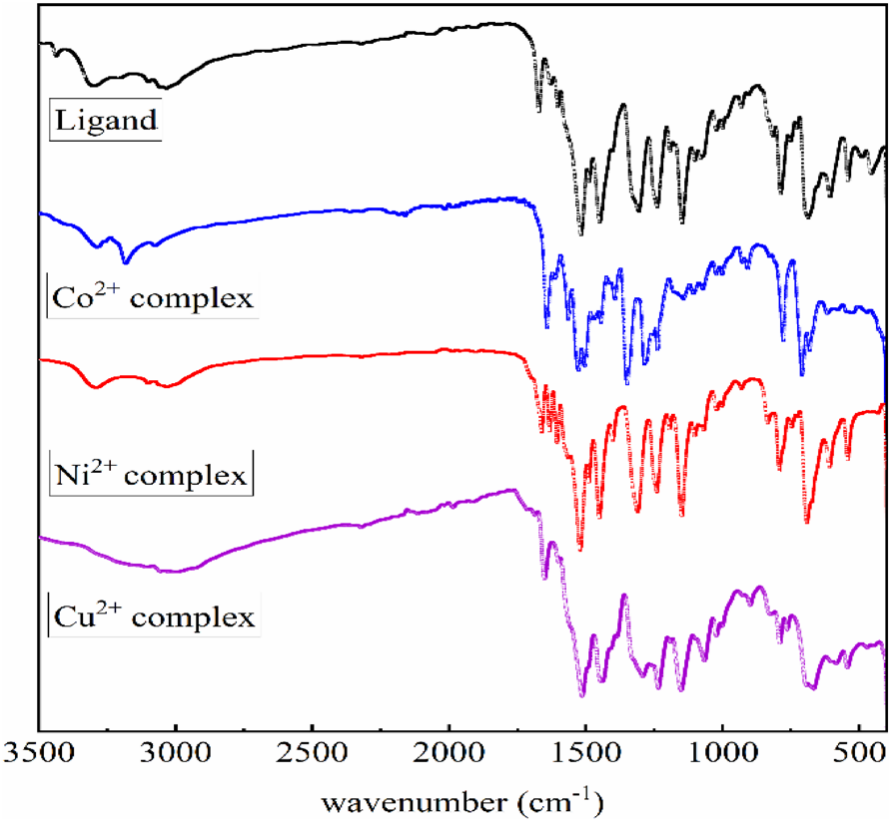
FT-IR spectra of ligand and its metal chelates.

**Figure 3:**
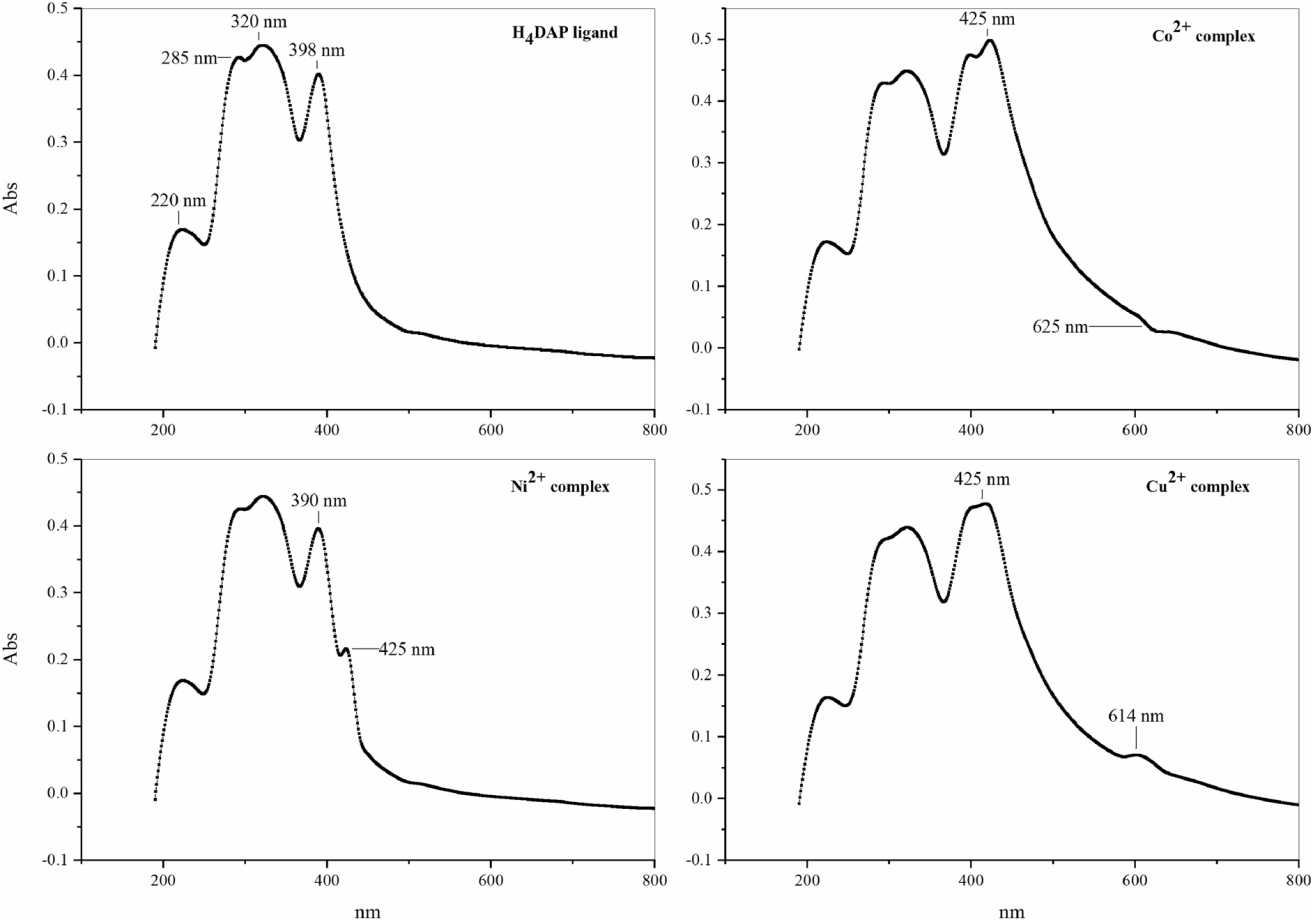
Electronic spectra of ligand and its metal chelates.

The structure of the ligand was confirmed via the following techniques:

- **FT-IR spectrum**: showed bands at 3316, 3052, and 1672 cm^−1^, assigned to υ(NH), υ(CH)_aromatic_, and υ(C=O), respectively. The band at 1251, and 731 cm^−1^ is equivalent to υ(C = S). The band at 1600 cm^−1^ characterized υ(C=N)_pyridine_ (Fig. 2).
- ^**1**^**H- and **^**13**^**C-NMR and spectrum (**Figure S5**)**: the ^1^HNMR spectrum provides additional proof of the hypothesized design of the ligand. The ^1^HNMR spectrum was acquired in DMSO-d_6_ with respect to TMS. The ^1^HNMR spectral data displayed signals at (δ ppm): 6.28 [m, py-2H^(32,33)^], 7.41 [m, py-1H^(31)^], 7.41–7.52 [m, ph-4H^(35,37, 40,42)^], 7.62 [m, ph-2H^(36,41)^], 7.91–8.00 [m, ph-4H^(34,38, 39,43)^], 11.51 [s, 2H, NH^(44,47)^], 12.98 [s, 2H, NH^(45,46)^], while the ^13^CNMR spectrum showed signals at 168.52, 176.19, and 149.98 ppm corresponding to carbon atoms of (–C_23, 29_=O), (–C_20, 26_=S), and (–C_4, 2_–N), respectively, and the other aromatic carbon signals were detected at 99.98, 128.55, 128.80, 132.14, 133.35, and 139.83 ppm.
- **Electronic spectrum**: the UV–vis spectrum of H_4_DAP (1 × 10^−4^M/DMSO) displays four absorption bands, the first of which is 223 nm is assigned for π–π* transition within the aromatic system. The second (293 nm) and third bands (322 nm) involve π–π* transitions in C=N, C=S, and C=O. The longer wavelength band at 398 nm can be assigned to intramolecular CT interaction within the whole molecule (Fig. 3).

#### Characterization of the isolated metal complexes

The structures of the metal complexes were established by the subsequent techniques:

- **Molar conductance measurements**: The molar conductivity values (Ω_m_) of the metal complexes (10^–4^ M/DMSO) at ambient temperature revealed that all the separated complexes provide Ω_m_ readings between 8.24 and 14.32 Ω^−1^ cm^2^ mol^−1^, which corroborated the non-electrolytic character of these complexes.
- **FT-IR spectra**: Each complex exhibited a wide spectrum at 3427–3372 cm^−1^ attributed to υ(OH) of coordinated and/or linked H_2_O molecules associated with the complexes and validated by elemental and thermal examination (Fig. 2). The unique band due to υ(C=O), which was noticed at 1672 cm^−1^ in the FT-IR spectrum of the free ligand, demonstrated apparent shifts to a lower wavenumber (1656–1653 cm^−1^) in the FT-IR spectra of Ni^2+^ and Cu^2+^ complexes, suggesting the role of the oxygen atom of the group C=O in coordinating with the metal ions, on contrary, this band vanished in the FT-IR spectra of the Co^2+^ complex with the appearance of newly formed bands at 1643 cm^−1^ owing to the creation of new C=N, verifying coordination of the ligand with the deprotonated OH group. The peak of υ(C=S), which occurred at 1251 cm^−1^ in the FT-IR spectrum of the unbound ligand, altered to a lower level (1236 cm^−1^) in the FT-IR spectra of the two Co^2+^ and Cu^2+^ complexes, suggesting the interaction of the S atom with the metal ions. Whereas in the Ni^2+^ complex, the band of υ(C=S) faded and a new band at 1630 cm^−1^ developed due to the freshly generated C=N group. The distinctive vibration that characterizes (C=N)_pyridine_ was barely influenced by the complexation, hence ignoring the probability of its attaching to the metal ions. Evidence for the above view is the creation of new bands at 551–510 and 459–409 cm^−1^ in the FT-IR spectra that might be attributed to υ(M–O) and υ(M–N)^34^.
- **Electronic spectra and magnetic moment measurements**: The UV–Vis spectrum of the Co^2+^ complex displays prominent peaks at 220, 285, 320, and 398 nm as a result of the ligand field. The absorption bands at 425 nm are associated with the ^4^T_1_g^(F)^ → ^4^T_1_g^(P)^ transition, while the band at 625 nm can be related to the ^4^T_1_g^(F)^ → ^4^A_1_g^(F)^ transition. These transitions match tetrahedral Co^2+^ complexes^35^. The magnetism of the Co^2+^ complex was determined to be 3.71 B.M. This result is indicative of the existence of three unpaired electrons in d-orbitals, which confirms the tetrahedral geometry. The absorption at 425 nm seen in the Ni^2+^ complex can be attributed to the ^3^T_1_ → ^3^T_1_(P) transition in a tetrahedral structure^36^. The magnetic moment of the Ni^2+^ complex is 2.73 B.M., which aligns with the reported value for a d^8^-tetrahedral shape. However, the Cu^2+^ complex exhibits ligand field absorption bands as well as a broad band at 614 nm. This absorption band can be attributed to the ^2^B_1_g → ^2^Eg transition in an octahedral structure^37^. The complex has a magnetic moment of 1.75 B.M., which suggests the presence of an octahedral geometry surrounding the Cu^2+^ ion (Fig. 3).

#### Thermal gravimetric analysis and kinetic data

**Figure 4:**
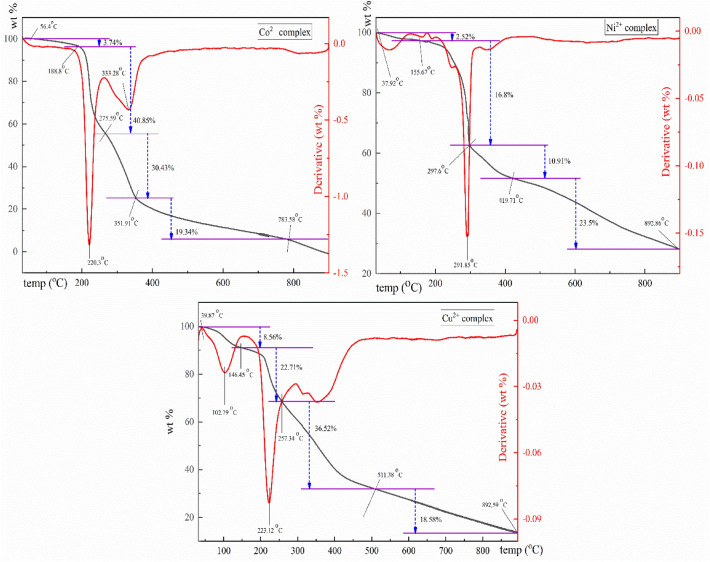
TG-DTG curves and temperature ranges of the isolated metal complexes.

Thermal degradation patterns were examined under N_2_ flow to verify the chemical formula. The TG-DTG curves are given in Fig. 4, and ranges of temperatures, mass losses, and decomposition procedures are provided in Table 1. From TG curves, Co^2+^, Ni^2+^, and Cu^2+^ complexes lose 94.36%, 53.73%, and 86.37% of their mass. The TG curves demonstrated that the thermal breakdown of metallic complexes experiences four degradation steps for Co^2+^, Ni^2+^, and Cu^2+^ complexes removing distinct fragments at temperatures ranging from 30 to 900 °C. The breakdown pattern of metal complexes demonstrates the division of chelates and the rendering of corresponding metal oxides at maximum temperature.

**Table 1:** Kinetic parameters attained by Eyring equation for the metal complexes.

Complex	Step	TG range (°C)	Mass loss %	E_a_ KJ\mol	A (S^−1^)	∆H* KJ\mol	∆S* KJ\mol.K	∆G* KJ\mol
Co^2+^ complex	1st	56.4–188.8	3.74	31.02	36.30	27.71	− 0.2175	114.44
	2nd	188.8–275.59	40.85	97.40	1.05 × 10^8^	93.13	− 0.0958	142.32
	3rd	275.59–351.91	30.43	135.59	7.70 × 10^9^	130.45	− 0.0614	167.08
	4th	351.91–783.59	19.34	54.41	38.70	48.06	− 0.2226	224.05
Ni^2+^ complex	1st	37.92–155.67	2.52	21.85	5.73	18.44	− 0.2331	114.15
	2nd	155.67–297.6	16.8	79.53	3.24 × 10^5^	75.29	− 0.1439	148.63
	3rd	297.6–419.71	10.91	81.26	3.13 × 10^4^	75.95	− 0.1652	181.61
	4th	419.71–892.86	23.5	113.28	2.21 × 10^5^	106.46	− 0.1510	230.25
Cu^2+^ complex	1st	39.87–146.45	8.56	49.00	4.91 × 10^4^	45.90	− 0.1570	104.44
	2nd	146.45–257.34	22.71	96.09	1.01 × 10^8^	92.05	− 0.0958	138.61
	3rd	257.34–511.38	36.52	59.79	4.18 × 10^2^	54.60	− 0.2009	180.03
	4th	511.38–892.59	18.58	113.61	1.99 × 10^4^	106.30	− 0.1716	257.23

*Thermodynamic parameters of activation.

The Co^2+^ complex shows a loss of one water molecule from 56.4 to 188.8 °C and then degrades in three steps, accompanied by a 90.62% mass loss, leaving 5.64% residue. The second and third steps occurred at a maximum temperature of 220.3 °C and 333.28 °C, respectively. On the other hand, the first stage of degradation for the Ni^2+^ complex occurred between 37.92 and 155.67 °C, which was accompanied by mass losses of 2.52%. This step corresponds to the loss of one H_2_O molecule in the Ni^2+^ complex. The second decomposition step occurred between 155.67 °C and 297.6 °C (maximum temperature of 291.85 °C) and was associated with 16.8% weight loss. And the third decomposition step within the range of 297.6–419.71 °C is accompanied by mass losses of 10.91%. The fourth decomposition step occurs at temperatures of 419.71–892.86 °C. For the Cu^2+^ complex; the first degradation step occurs from 39.87 °C to 146.45 °C, accompanied by mass losses of 8.56%, corresponding to the loss of three H_2_O molecules. The second stage is at peak temperatures of 223.12 °C (from 146.45 °C to 257.34 °C), while the third degradation step in a range of 257.34–511.38 °C is associated with a mass loss of 36.52%, and the final decomposition step takes place at temperatures of 511.38–892.59 °C.

The kinetic variables, Arrhenius pre-exponential factor (A), and activation energy (Ea) of the degradation phases were computed using the Coats-Redfern approach^37^. Furthermore, the thermodynamic variables entropy (∆S*), enthalpy (∆H*), and Gibbs free energy (∆G*) (Table 1) were computed employing the Eyring equation^37^. The Cu^2+^ complex, as an illustration, revealed that the first stage of breakdown occurred at its highest temperature of 102.79 °C, which correlates with the elimination of three uncoordinated H_2_O molecules, producing Ea of 49.00 kJ/mol, ΔH* of 45.90 kJ/mol, and ΔG* of 104.44 kJ/mol. Additionally, the complex experiences a second breakdown and gives a break at a peak temperature of 223.12 °C with Ea of 96.09 kJ/mol, ΔH* of 92.05 kJ/mol, and ΔG* of 138.61 kJ/mol. Consequently, the complex displayed a progressive disintegration from 275.34 to 511.38 in a third breakdown step with Ea of 59.79 kJ/mol, ΔH* of 54.60 kJ/mol, and ΔG* of 180.03 kJ/mol. Likewise, the last stage of breakdown happened from 511.38 °C to 892.59 °C with an Ea of 113.61 kJ/mol, ΔH* of 106.30 kJ/mol and ΔG* of 257.23 kJ/mol.

#### Powder X-ray diffraction studies

The crystal structures of polymorphs for the isolated compounds were determined using the PXRD pattern, employing the Reflex module within BIOVIA MS software. Various modules, including DMoL^3^, TREOR90, Powder Solve, Pawley Refinement, and Rietveld Refinement, were utilized in this process^38^. Geometry optimization was conducted using the GGA/RPBE functional/DNP basis set. Peak indexing was performed in the range of 5°–80°, and the resulting unit cell from indexing was employed for Pawley refinement. The R_wp_ value obtained played a crucial role in establishing the relationship between experimental and simulated PXRD. Subsequently, the optimized structure of the molecules and the generated unit cell underwent Reflex Powder Solve, incorporating a simulated annealing algorithm. Further refinement of the structure was carried out through Rietveld refinement, leading to the final structure solution (Figure S6). The unit cells, together with other lattice parameters for the ligand and its complexes, are reported in Table S1. The analysis of the obtained data indicates distinct PXRD patterns for all compounds, confirming the successful coordination of metals with the ligand to produce the complex compounds. The average grain sizes of the isolated compounds were determined to be 29.12 nm, 34.40 nm, 20.16 nm, and 39.62 nm for the H_4_DAP ligand, Co^2+^, Ni^2+^, and Cu^2+^ complexes, respectively. This suggests that the compounds exist at the nanoscale.

#### TEM and SEM analysis

TEM micrographs of the H_4_DAP ligand are shown in Figure S7, which has a flake-like morphology. The size of the ligand particles was measured using ImageJ software. The average size of the flake-like H_4_DAP particles is 26.75 nm. A micromorphological study of H_4_DAP ligand by SEM (Figure S7) shows irregular grain sizes with distinct separations from each other.

### QM computations

#### Geometry optimization

The best configurations of the isolated compounds, lengths of bonds, and angles are compiled and demonstrated in Figure S8. It was revealed that the arrangement of Co^2+^ and Ni^2+^ complexes is deformed tetrahedral. This distortion is related to the bulky substituents surrounding the metal ions, which create noticeable distortion in the structures. Furthermore, a distorted octahedral Cu^2+^ geometrical form is observed because of the values of the bond angles involved. The deformation effect around metal ions could be due to the pyridine and aromatic phenyl rings of the ligand that surround the coordination sphere; this may give additional steric in binding, resulting in a shift in the donating atom position^39,40^.

#### Quantum chemical reactivity parameters

The ability of the Schiff base to create stable complexes with various transition metals stems from the presence of atoms in its vicinity with elevated electron density. These atoms serve as coordination sites for the transition metal, facilitating the establishment of covalent interactions between the ligand and the metallic ion. Simultaneously, these interactions involve a partial sharing of the positive charge associated with the metallic ion^41^. These interactions are enabled by peripheral molecular orbitals, where the HOMO of the ligand gives energized electrons to the LUMO of the metal. Upon examining the isosurface of the HOMO and its projection onto the total charge density surface, it was observed that four metallic ion binding centers are in the H_4_DAP ligand. These binding centers contain the two oxygen atoms (two C=O) and the two sulfur atoms from the C=S groups. Table S2 displays the quantum reactivity parameters for both the ligand and complexes. Figure S9 shows the electron transition map (HOMO → LUMO) for the studied complexes. The second derivative of energy (hardness) provides insights into the stability and reactivity of a molecule. In comparison to global hardness (H), global softness (S) represents its inverse. Large ΔE forecasts the hardness of molecules exhibiting low polarizability and strong kinetic vulnerability, while small hardness is associated with the opposite.

#### Molecular electrostatic potential (MEP)

The MEP maps serve as an indicator of electronic density, aiding in the identification of sites susceptible to electrophilic attack, nucleophilic reactions, and hydrogen bonding interactions. Analyzing the MEP map of the ligand reveals a prominent negative region concentrated around oxygen and sulfur atoms, characterized by the highest intensity of the red color. This intensity arises from the presence of lone-pair electrons on the oxygen and sulfur atoms, making them favorable sites for electrophilic attack. Conversely, positive potential sites (blue color) are observed around the hydrogen atoms on all sides, as illustrated in Figure S10.

### Topological analysis

#### QTAIM (quantum theory of atoms in molecule) analysis and NCI analysis

**Figure 5:**
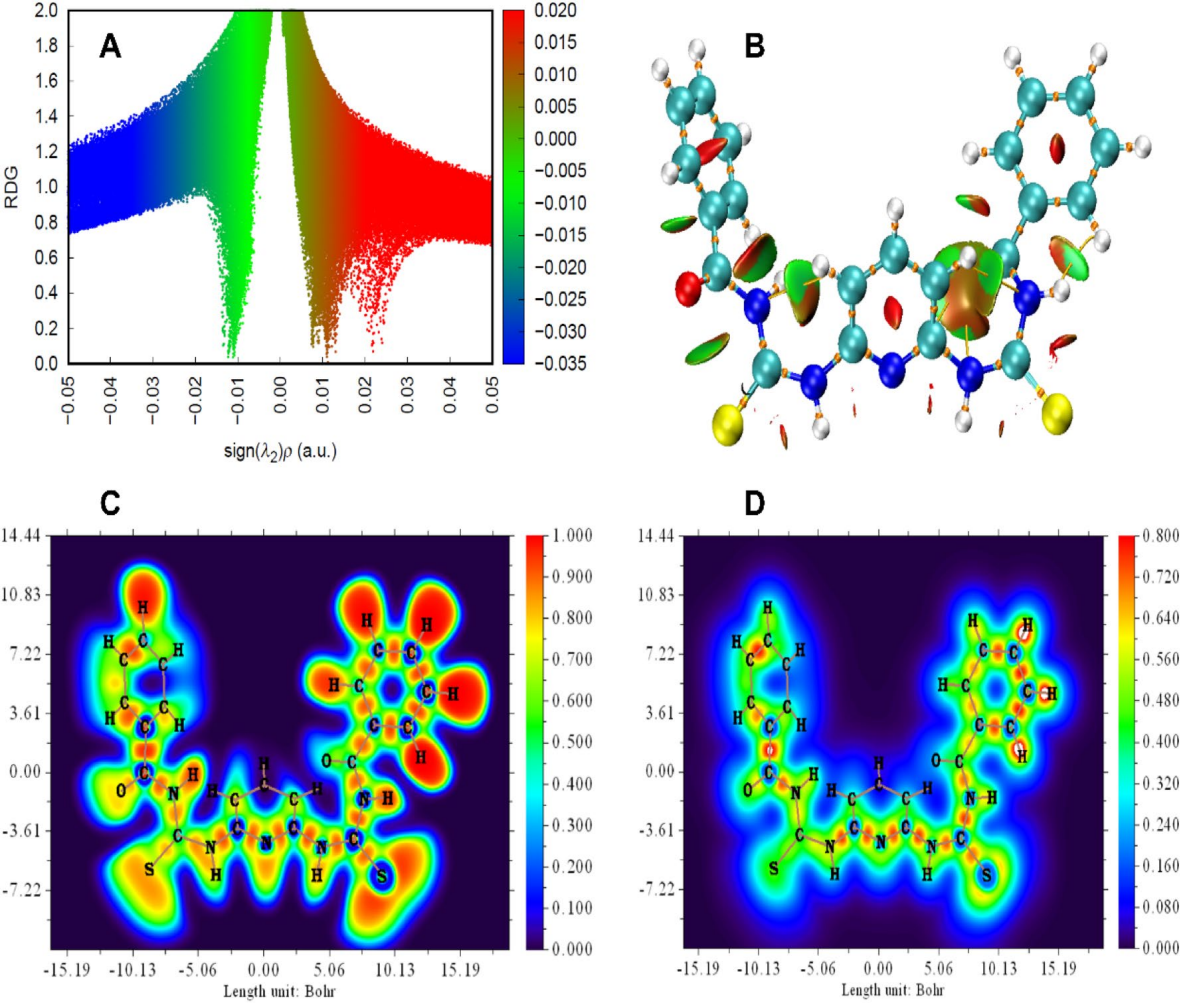
(**A**) RDG, (**B**) NCI, (**C**) ELF and (**D**) LOL colored filled maps of H_4_DAP ligand.

The QTAIM approach has been used to differentiate between intra- and intermolecular hydrogen bonding and to investigate the bond structure of molecular assemblies^42^. Examining the electron density topology of diverse intra- and intermolecular interactions provides a compelling means to validate the strength of these interactions. The nature of a hydrogen bond can be elucidated based on the following hypotheses: (i) strong hydrogen bonds are associated with values of ∇^2^(r) < 0 and H < 0, (ii) intermediate-form hydrogen bonds are indicated by values of ∇^2^(r) > 0 and H < 0, and (iii) weak hydrogen bonds are characterized by values of ∇^2^(r) > 0 and H > 0^43^. The intensity of a hydrogen bond can be further characterized by assessing the hydrogen bond energy (E_HB_). This energy can be determined through the equation E_HB_ = ½ V_BCP_. Recognizing the limitations of the QTAIM analysis in identifying all anticipated weak non-covalent interactions, particularly intramolecular hydrogen bonds, the NCI approach was employed as an additional method^44^. This study facilitates the visualization of regions involved in either repulsive or attractive interactions. In the examined system, the existence of a non-covalent connection is indicated by characteristic spikes on scatter plots of s(r) versus ρ(r) in low-density and low-gradient areas. These spikes are not observed in the absence of covalent connections. Furthermore, considering the sign of λ_2_ (the second eigenvalue) of the Hessian matrix of electron density, valuable insights may be obtained on the nature of the noncovalent interaction. Specifically, a negative value of λ_2_ indicates a stabilizing effect, whereas a positive value of λ_2_ suggests a destabilizing effect. Therefore, the existence of a sharp increase in the low-density, low-gradient area with a negative λ_2_ suggests a stable interaction, including a hydrogen bond. Conversely, a smaller spike with a moderately negative λ_2_ reflects a weakly stabilizing relationship. Lastly, when a spike coincides with a positive λ_2_, it shows the lack of a non-covalent connection. The isosurfaces of the s(r) were studied, and corresponding plots were obtained for both the H_4_DAP ligand and its complexes, as represented in Figs. 5 and S11. In these figures, the intramolecular H-bond was detected energetically in a range of − 0.05 to − 0.02 a.u., whereas the VDW interaction was energetically positioned between − 0.015 and 0.005 a.u. Additionally, the area suggestive of a substantial steric effect was defined by two spikes at 0.01 and 0.005 a.u. (Table S3).

#### ELF and LOL

The color-filled maps of ELF (Electron Localization Function) and LOL (Localized Orbital Locator) illustrate localized electron density regions and molecular orbitals, revealing covalent bond interactions in a molecule^45^. The highest values of ELF range between 0.5 and 1.0 Bohr for all substances. These values signify tightly confined portions of the molecule. Conversely, the lowest value suggests a significantly delocalized region of electrons inside the molecule. The electron density exhibiting delocalization is situated over the C, N, and S of the ligand. Utilizing the LOL map, the spatial distribution of localized and delocalized molecular orbitals in the examined compounds is elucidated. A color-filled map of LOL for the compounds is generated using a range from 0.0 to 0.8 Bohr. The n-delocalized orbital is depicted in blue, primarily centered around the C, N, and S atoms. In contrast, the n-localized ones are in red, predominantly associated with the H atoms (Fig. 5).

### Florescence spectral analysis

**Figure 6:**
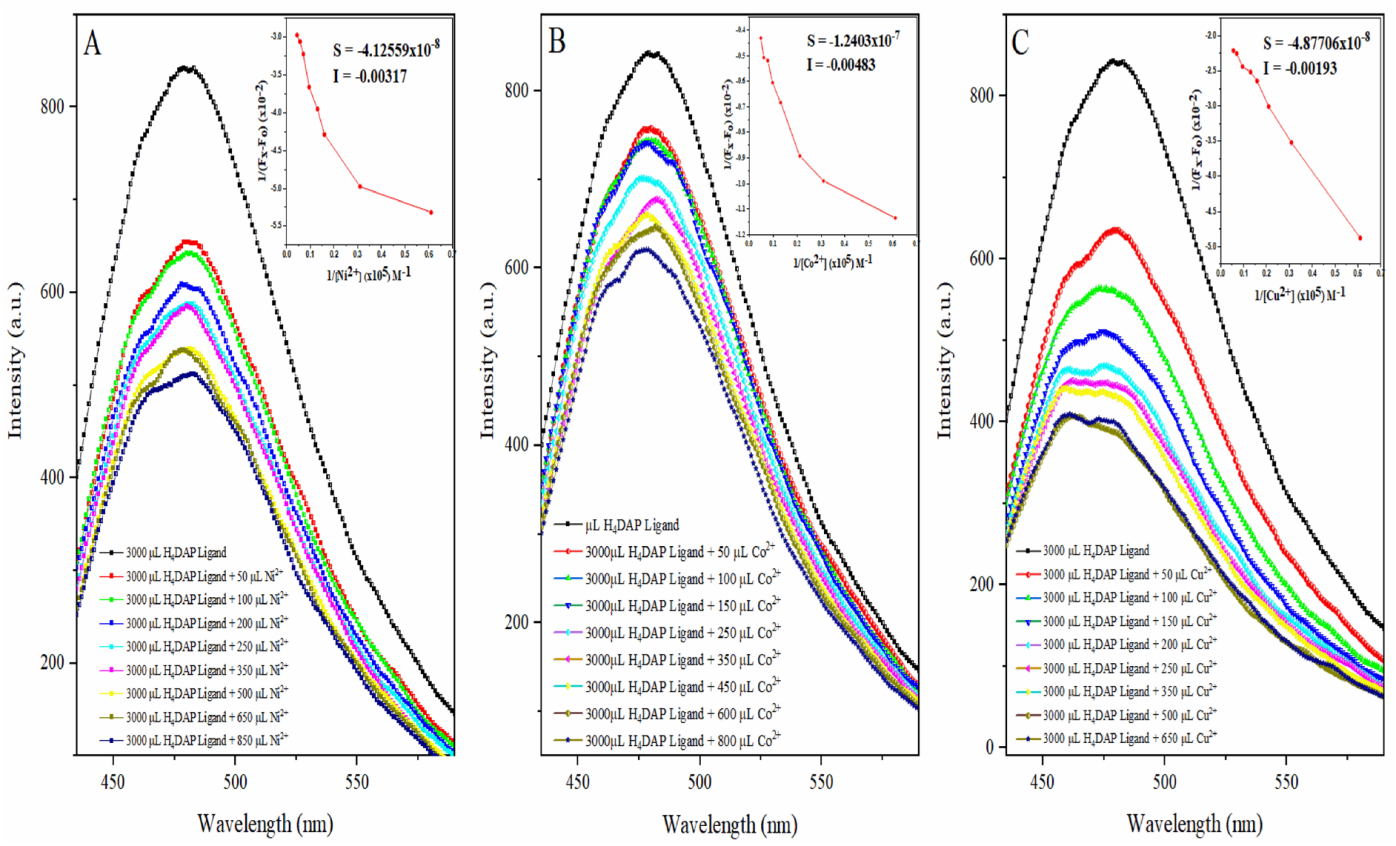
Fluorescence emission spectra of H_4_DAP recorded in the absence and presence of escalating concentrations of M^2+^, and Benesi-Hildebrand plots.

Fluorescence emission spectroscopy serves as a versatile biophysical technique employed for investigating the binding mechanism of ligand–metal interactions and assessing associated binding parameters^46,47^. The metal-binding potential of the H_4_DAP ligand was assessed by examining its optical properties through emission spectroscopy in the presence of Co^2+^, Ni^2+^, and Cu^2+^ metal ions. Stock solutions of H_4_DAP ligand, CoCl_2_, NiCl_2_.6H_2_O, and CuCl_2_.6H_2_O salts were prepared in DMSO in 1.0 × 10^−3^ M concentrations. The fluorescence emission spectra of H_4_DAP ligand with different additions of Co^2+^, Ni^2+^, and Cu^2+^ metal ions are given in Fig. 6. It is apparent from this that H_4_DAP ligand exhibits a strong emission peak at 481 nm upon excitation at 292 nm. Further, for the interaction of the investigated ligand with metal ions in DMSO, the intensity of the band was lowered (quenching), especially in the case of the addition of Cu^2+^ metal, and the quenching increased with the increase in metal ion concentration. The rationale behind this conclusion can be elucidated by considering the paramagnetic characteristics of the metal ions, wherein the singlets and triplets of the ligand are effectively suppressed by the presence of unpaired d-orbital electrons^37^. The observed quenching implies the creation of the H_4_DAP-M^2+^ system involving the ligand and metal ions through the sulfur and oxygen atoms, indicating a potential alteration in the microenvironment of H_4_DAP upon interaction with metal cations.

Fluorescence data were employed to calculate the binding constant (K_b_) of the L-M system utilizing Eq. (1)^48^:

1 $$\frac{1}{{F}_{x}-{F}_{o}}=\frac{1}{{F}_{\infty }-{F}_{o}}+\frac{1}{{K}_{b}{\Delta F}_{max}[{M}^{2+}]}$$

In the given equation, F_o_, F_x_, and F_∞_ represent the emission intensities of the H_4_DAP ligand under different conditions: in the absence of metal ions, at specific concentrations of metal ions, and at the concentration of complete interaction, respectively. [M]^2+^ denotes the concentration of metal ions, and K_b_ stands for the binding constant for the interaction. The determination of the binding constant involved utilizing the slope and intercept obtained from the plot of $\frac{1}{{F}_{x}-{F}_{o}}$ against $\frac{1}{[{M}^{2+}]}$. The resulting values were determined to be 1.76 × 10^4^, 3.62 × 10^4^, and 3.96 × 10^4^ M^−1^ for Co^2+^, Ni^2+^, and Cu^2+^, respectively, indicating a moderate binding affinity between the ligand and metal ions.

### Biological activity and molecular docking

#### Antibacterial and antifungal activities

The obtained results are presented in Table 2, which show that:

- The ligand exhibits moderate activity against *A. fumigatus*, *S. aureus*, and *E. coli*, while showing no activity against *C. albicans*,* B. subtilis*, and *P. vulgaris*.
- The Ni^2+^ complex demonstrates activity against all microbial strains, surpassing the efficacy of ketoconazole and gentamycin, particularly against *A. fumigatus*,* C. albicans*, and *P. vulgaris*.
- The Cu^2+^ complex is active solely against *C. albicans*,* B. subtilis*, and* E. coli.*
- The Co^2+^ complex displays inactivity against all microorganisms except for activity against *C. albicans* and *E. coli*.

**Table 2:** Results include the mean zone of inhibition measured in millimeters observed against various pathogenic microorganisms.

Organism		Tested Compound				Control	
		H_2_DAP ligand	Co^2+^ complex	Ni^2+^ complex	Cu^2+^ complex	Ketoconazole	Gentamycin
Fungi	*A. fumigatus*	12	–	36	–	17	–
	*C. albicans*	–	13	30	15	20	–
Gram+	*S. aureus*	10	–	19	–	–	24
	*B. subtilis*	–	–	26	15	–	26
Gram−	*E. coli*	14	13	25	13	–	30
	*P. vulgaris*	–	–	34	–	–	25

The enhanced activity of the Ni^2+^ complex is attributed to the inhibition of bacterial growth through the interaction of transition metals with thiol groups (–SH) in enzymes, leading to the deactivation of these enzymes. Furthermore, the increase in lipophilicity induced by the metal ion complexation contributes to the permeation of the lipid film in the microbial cell membrane, amplifying the overall antimicrobial effect^49^. The variations in the activity of the metal complexes against different strains are contingent upon factors such as penetrability and potential differences in the ribosomes of microbial cells. These differences are also influenced by the geometry and types of groups linked to the metal ion^50^. The ineffectiveness of certain compounds against particular strains is attributed to either the functioning of efflux pumps or the obstacles posed by these microbes^50^.

#### Cytotoxic assay

**Figure 7:**
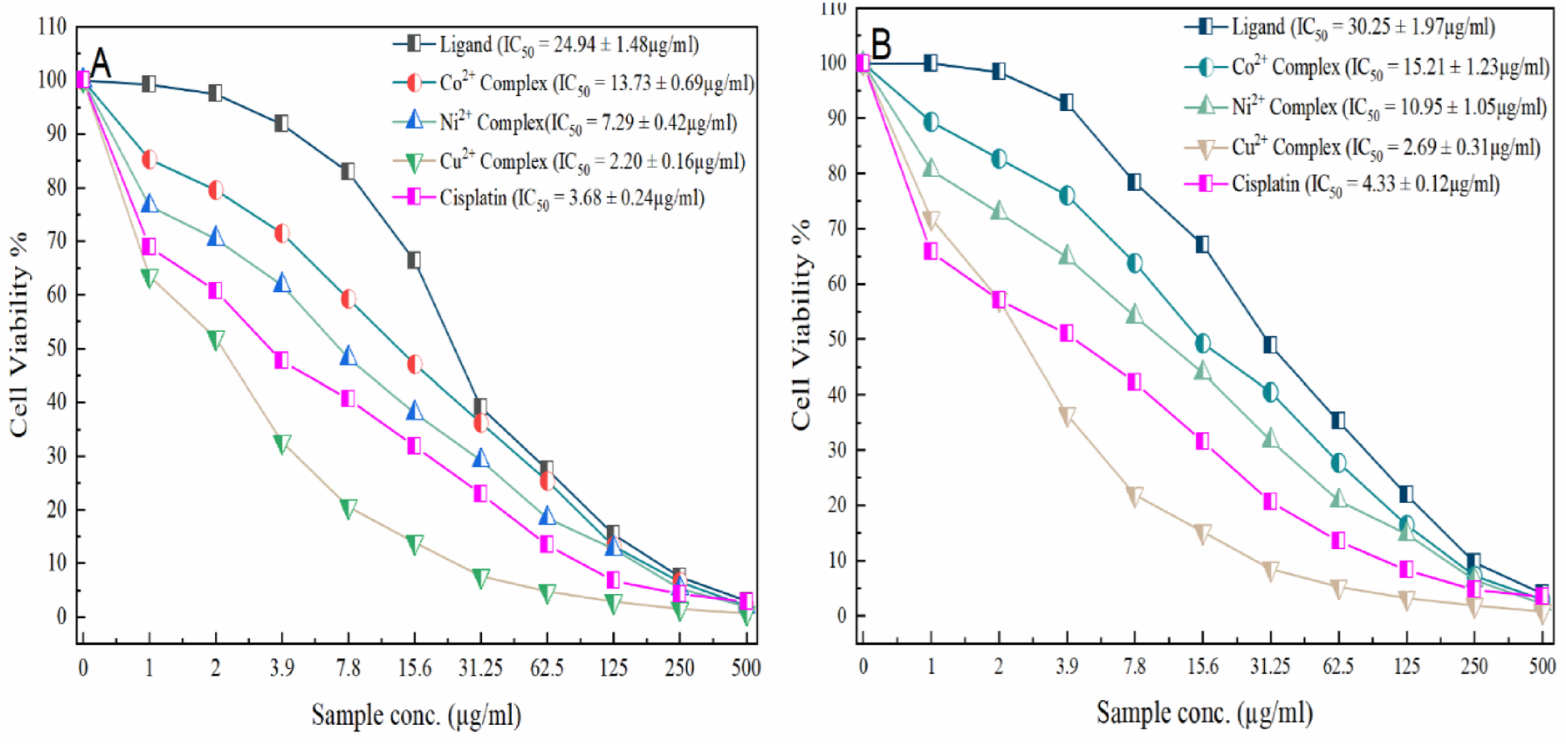
Cytotoxic activity and IC_50_ values for ligand and its complexes using (**A**) MTT, and (**B**) crystal violet assays.

The H_4_DAP ligand, along with the Co^2+^, Ni^2+^, and Cu^2+^ complexes, underwent in vitro cytotoxicity testing against HEPG-2 and MDA-MB-231 cell lines using the MTT assay across a range of concentrations (1–500 μg/ml). Figure 7 illustrates the cell viability (%) plotted against concentrations obtained through continuous exposure. Cisplatin was employed as the control in this comparison.

The concentration-dependent cytotoxicity of the complexes was established. Screening outcomes revealed significant anticancer activity for all metal complexes. The cytotoxic effects at a low concentration (1 μg/ml) were organized for the drugs against HEPG-2 cell lines as follows: Cu^2+^ complex (36.52% ± 0.94) > Ni^2+^ complex (23.42% ± 0.64) > Co^2+^ complex (14.69% ± 0.73) > H_4_DAP ligand (0.77% ± 0.12). At a concentration of 500 μg/ml (high dose), the activities are organized as follows: Cu^2+^ complex (99.37% ± 0.19) > Ni^2+^ complex (98.04% ± 0.42) > Co^2+^ complex (97.96% ± 0.08) > H_4_DAP ligand (97.03% ± 0.19). Additionally, the Cu^2+^ complex showed the highest inhibition against the MDA-MB-231 cell line (28.16% for the low dose and 99.25% for the high dose), while the H_4_DAP ligand displayed the lowest activity (0% for the low dose and 95.99% for the high dose). The NSO-donor ligand utilized in the prepared complexes heightens the reactivity of metal ions, resulting in increased biological activity compared to the metal salts.

The complexes exhibit a heightened ability to damage DNA compared to the non-coordinated ligand. The planar aromatic rings enable the complex to approach DNA more closely through intercalation between adjacent base pairs of DNA chains, forming π-stacking bonds with them^51^. The complexes engage with the DNA molecule through intercalation within the double helix structure. Subsequently, the M^2+^ ion undergoes reduction to the M^+^ ion, and in the presence of O_2_, the resulting M^+^ complex generates reactive oxygen species in proximity to the double helix. These species then initiate an attack on the 2-deoxyribose moiety, resulting in the cleavage of the DNA chain^52^.

#### Antioxidant activity

##### ABTS free radical scavenging activity

ABTS (2,2'-azinobis-3-ethylbenzothiazoline-6-sulfonic acid) exhibits a characteristic absorbance peak at 734 nm, which diminishes as the proton radical is scavenged. The ligand and its metal complexes were subjected to antioxidant activity testing at various concentrations using the ABTS assay (Figure S12). The observed activities were then compared to those of L-ascorbic acid, which serves as a standard drug. The Co^2+^ complex showed the best radical scavenging activity (for 1000 µg/ml concentration), with 95.17% scavenging (IC_50_ = 6.001 ± 0.38 µg/ml), followed by the Cu^2+^ complex, which shows 85.21% ABTS scavenging activity (IC_50_ = 32.23 ± 4.05 µg/ml). The Ni^2+^ complex and H_4_DAP ligand show the lowest activity with 76.81% (IC_50_ = 87.37 ± 6.21 µg/ml) and 71.43% (IC_50_ = 190.57 ± 7.64 µg/ml).

##### DPPH scavenging

The H_4_DAP ligand and its metal complexes were assessed for DPPH radical scavenging activity (Figure S12). Notably, all the complexes exhibited significant antioxidant potential, as indicated by their IC50 values, 113.65 ± 5.23, 3.38 ± 0.13, 58.76 ± 4.16, and 25.31 ± 2.45 μg/ml for H_4_DAP, Co^2+^, Ni^2+^, and Cu^2+^ complexes, respectively. The Co^2+^ showed better activities than standard ascorbic acid (IC_50_ = 10.21 ± 0.77 μg/ml). The ligand (IC_50_ = 113.65 ± 5.23 μg/ml) was found to be less active than standard as well as its respective complexes. Nickel (IC_50_ = 58.76 ± 4.16 μg/ml) and copper (IC_50_ = 25.31 ± 2.45 μg/ml) complexes demonstrated noteworthy antioxidant capabilities, indicating remarkable antioxidant activity. Therefore, the order of activity for these complexes is as follows: Co^2+^ > Cu^2+^ > Ni^2+^ > H_4_DAP ligand. The substantial activity exhibited by the complexes can be attributed to the stabilization of free radicals through the interaction of the metal ion with the phenolic moiety^53^.

##### Ferric reducing antioxidant power (FRAP)

The ability of the extract to reduce ferric ions to ferrous ions serves as an indicator of its potential antioxidant properties. An inspection of the data indicates that the Co^2+^ complex had the highest antioxidant activity, with 91.56 ± 0.24% inhibition scavenging at 1000 µg/ml concentration and an IC_50_ of 7.35 ± 0.35 µg/ml (Figure S12). The notable antioxidant capacity observed could be attributed to its distinctive coordination. These compounds emerge as a crucial category of promising antioxidants, potentially contributing to mitigating oxidative stress and providing protection against the detrimental effects of reactive oxygen species. While the Cu^2+^ complex of the H_4_DAP ligand showed moderate activity with an inhibition of 81% and an IC_50_ of 59.14 ± 5.28 µg/ml, on the other hand, lower antioxidant activity (73.76 and 67.29%) was corresponding to the Ni^2+^ complex and H_4_DAP ligand, with IC50 = 236.40 ± 9.17 µg/ml and 116.62 ± 7.13 µg/ml, respectively.

#### Molecular docking

In recent times, various small molecules have been identified as inhibitors of Bcl-2. The capacity of these molecules to inhibit the antiapoptotic Bcl-2 protein has been linked to the sensitization of cancer cells to apoptosis^54^. The inhibitors operated by binding to the binding groove in Bcl-2, thereby inhibiting its antiapoptotic effect. This paper presents a comparative molecular docking study conducted to evaluate the modes, binding affinities, and interactions of the compounds against a specific Bcl-2 inhibitor.

The redocking of the DRO ligand into Bcl-2 using MOE software demonstrated a binding free energy of − 8.22 kcal/mol for the most favorable conformation of DRO, as indicated by the results of this validation. The investigation into DRO's binding mode showed alignment with the co-crystallized ligand, displaying an RMSD of 2.07 Å. The analysis identified one hydrogen-donor, one ionic, and two pi-hydrogen interactions between DRO and residues GLU 111, PHE 63, and GLY 104 (Figure S13).

Docking scores and binding interactions resulting from the docking calculations of H_4_DAP ligand and its derivatives with the 2W3L target are presented in Table 3. It is evident from this table that the most favorable docking scores, determined by binding free energy, were: − 6.54, − 6.12, − 6.29, and − 5.99 kcal/mol for H_4_DAP ligand, Co^2+^, Ni^2+^, and Cu^2+^ complexes, respectively. H_4_DAP ligand showed five H-acceptor interactions between S 22, O 24, O 30, and LYS 22 and ARG 26, and ARG 66 with a binding energy of − 6.54 kcal/mol. The observed binding mode for the Co^2+^ complex indicates that it binds within the binding pocket, yielding a molecular docking score of − 6.12 kcal/mol with a Pi-H interaction observed between the 6-ring and VAL 115 residues (distance = 4.51 Å). The Ni^2+^ complex forms two H-donor interactions, existing between O 50 and ASP 61 (OD1) and SER 64 (OG) residues with distances of 2.78 and 2.79 Å, respectively. The Cu^2+^ complex has an energy of − 5.99 kcal/mol and is involved in making a pi-H interaction between the 6-ring and CE1 of the PHE 63 residue (4.61 Å).

**Table 3:** docking scores, type and length of bonds between compounds and residues of active sites.

Compound	S-score (Kcal/mol)	Interactions occurring between atoms of compounds and residues within the active site					
		Atoms within compound	Participating receptor atom(s)	Participating receptor residue(s)	Nature of interaction bond	Distance (Å)	Energies (Kcal/mol)
H_4_DAP	− 6.54	S22	CE	LYS22	H-acceptor	3.66	− 0.8
		S22	NH2	ARG26	H-acceptor	4.44	− 2.0
		O24	NH1	ARG26	H-acceptor	2.84	− 5.5
		O24	NH2	ARG26	H-acceptor	3.10	− 2.2
		O30	NE	ARG66	H-acceptor	3.12	− 4.3
Co^2+^ complex	− 6.12	6-ring	CG2	VAL115	Pi-H	4.51	− 0.5
Ni^2+^ complex	− 6.29	O50	OD1	ASP61	H-donor	2.78	− 5.4
		O50	OG	SER64	H-donor	2.79	− 2.6
Cu^2+^ complex	− 5.99	6-ring	CE1	PHE63	Pi-H	4.61	− 0.6
DRO reference	− 8.22	NAZ70	OE1	GLU111	H-donor	2.96	− 15.5
		NAZ70	OE1	GLU111	Ionic	2.96	− 4.8
		6-ring	CD1	PHE63	Pi-H	4.27	− 0.7
		6-ring	CA	GLY104	Pi-H	3.86	− 0.7

## Conclusion

In summary, metal complexes of the H_4_DAP ligand were successfully synthesized and characterized utilizing various techniques. The Cu^2+^ complex displayed an octahedral structure, while the Co^2+^ and Ni^2+^ complexes exhibited a tetrahedral geometry. All the isolated compounds proved to be stable, colored, and insoluble in water. The PXRD patterns suggested a semicrystalline structure for the ligand, Co^2+^, and Ni^2+^ complexes, and an amorphous structure for the Cu^2+^ complex. The results from SEM micrographs corroborated well with those calculated from the Debye–Scherrer equation, confirming the accuracy of our particle size. DFT and QTAIM calculations were employed to investigate the reactivity and bond structure of the molecular systems of the isolated compounds. The compounds were evaluated for antimicrobial activity against various microbial strains, showcasing significant efficacy in the Ni^2+^ complex. Additionally, their antioxidant potential was assessed through ABTS, DPPH, and FRAP assays, revealing the Co^2+^ complex to possess the lowest IC_50_. In terms of anticancer activity, the Cu^2+^ complex demonstrated the lowest IC_50_ values against both HEPG-2 and MDA-MB-231 cell lines. Molecular docking against Bcl-2 revealed negative scores for all tested compounds, indicating their potential as anticancer agents, further supported by comparisons with the docking score of the reference ligand DRO.

### Ethical approval

We have no financial conflicts or personal connections to declare. Also, this research did not involve the use of both humans and/ or animals.

### Supplementary Information

Supplementary Information.

## Data Availability

The authors declare that the data supporting the findings of this study are available within the paper and its Supplementary Information files. Should any raw data files be needed in another format they are available from the corresponding author upon reasonable request. Source data are provided with this paper.
