# Supplementary material for: Synthesis, characterization, biological potency, and molecular docking of Co2+, Ni2+ and Cu2+ complexes of a benzoyl isothiocyanate based ligand
Source: Sci Rep. 2024 May 1;14:10032. doi: 10.1038/s41598-024-58108-5 (PMC11063136; doi:10.1038/s41598-024-58108-5)
Supplement: Supplementary file 1 — Supplementary Information. [file 41598_2024_58108_MOESM1_ESM.pdf]

Synthesis, Characterization, Biological Potency, and Molecular Docking of Co^{2+}, Ni^{2+} and Cu^{2+} Complexes of a Benzoyl Isothiocyanate Based Ligand

Eida S. Al-Farraj[1], Adel M. Younis[2], Gaber M. Abu El-Reash[2*]

[1] Department of Chemistry, College of Science, Imam Mohammad Ibn Saud Islamic University (IMSIU), Riyadh, 11623, Saudi Arabia.

[2] Department of Chemistry, Faculty of Science, Mansoura University, Egypt.

[*] Corresponding author: Prof. Dr. Gaber M. Abu El-Reash: gaelreash@mans.edu.eg

1. Experimental

1.1. Antimicrobial Activity

The antimicrobial activities of the H_4DAP ligand and its complexes were investigated in laboratory conditions against specific types of fungi (Aspergillus fumigatus and Candida albicans), gram-positive bacteria (Staphylococcus aureus and Bacillus subtilis), and gram-negative bacteria (Proteus vulgaris and Escherichia coli) using established methods [26,27]. The tested microorganisms are thoroughly applied to the surfaces of Müller-Hinton Agar growing media in our experiment. Subsequently, the ligand and its complexes were made in amounts of 10 mg/ml, utilizing DMSO as the solvent. Holes were made in the plates of agar, and then prepared specimens of the isolated chemicals were inserted into the wells. All plates were stored at ambient temperature (35°C) for 24 h. The antimicrobial drugs diffused over the agar surface. Inhibition of germination and microbial expansion took place, and the diameter of the inhibition zones (mm) was measured.

1.2. Antitumor activity

1.2.1. MTT assay

- Mammalian cell lines: HepG-2 cells (human hepatocellular carcinoma) were obtained from the American Type Culture Collection (ATCC, Rockville, MD).
- Cell line propagation: The cells were grown on RPMI-1640 medium supplemented with 10% inactivated fetal calf serum and 50µg/ml gentamycin. The cells were maintained at 37ºC in a humidified atmosphere with 5% CO_2 and subcultured two to three times a week.
- Cytotoxicity evaluation using viability assay: For antitumor assays, the tumor cell lines were suspended in medium at a concentration of $5x10^4$ cells/well in 96-well tissue culture plates, then incubated for 24 hr. The tested compounds were then added into 96-well plates (three replicates)

to achieve different concentrations for each compound. Six vehicle controls with media or 0.5 % DMSO were run for each 96-well plate as a control. After incubating for 24 h, the number of viable cells was determined by the MTT test. Briefly, the media was removed from the 96-well plate and replaced with 100 µl of fresh culture RPMI 1640 medium without phenol red, followed by 10 µl of the 12 mM MTT stock solution (5 mg of MTT in 1 mL of PBS) in each well, including the untreated controls. The 96-well plates were then incubated at 37°C and 5% CO_2 for 4 hours. An 85 µl aliquot of the media was removed from the wells, and 50 µl of DMSO was added to each well, mixed thoroughly with the pipette, and incubated at 37°C for 10 min. Then, the optical density was measured at 590 nm with the microplate reader to determine the number of viable cells, and the percentage of viability was calculated as $[(OD_t/OD_c)]$ x 100%, where OD_t is the mean optical density of wells treated with the tested sample and OD_c is the mean optical density of untreated cells. The relation between surviving cells and drug concentration is plotted to get the survival curve of each tumor cell line after treatment with the specified compound. The 50% inhibitory concentration (IC_{50}), the concentration required to cause toxic effects in 50% of intact cells, was estimated from graphic plots of the dose response curve for each concentration [28-30].

1.2.2. Crystal violet assay

- Mammalian cell lines: MDA-MB-231 cells (breast carcinoma cells), were obtained from the VACSERA Tissue Culture Unit.
- Cell line propagation: The cells were propagated in Dulbecco's modified Eagle's medium (DMEM) supplemented with 10% heat-inactivated fetal bovine serum, 1% L-glutamine, HEPES buffer, and 50µg/ml gentamycin. All cells were maintained at 37ºC in a humidified atmosphere with 5% CO_2 and subcultured twice a week.
- Cytotoxicity evaluation using viability assay: For the cytotoxicity assay, the cells were seeded in a 96-well plate at a cell concentration of 1×10^4 cells per well in 100µl of growth medium. Fresh medium containing different concentrations of the test samples was added after 24 h of seeding. The microtiter plates were incubated at 37ºC in a humidified incubator with 5% CO_2 for a period of 24 h. Three wells were used for each concentration of the test samples. Control cells were incubated without test samples and with or without DMSO. The small percentage of DMSO present in the wells (maximum 0.1%) was found not to affect the experiment. After incubation of the cells at 37°C for 24 h, the viable cell yield was determined by a colorimetric

method. In brief, after the end of the incubation period, the media were aspirated, and the crystal violet solution (1%) was added to each well for at least 30 minutes. The stain was removed, and the plates were rinsed using tap water until all excess stain was removed. Glacial acetic acid (30%) was then added to all wells and mixed thoroughly, and then the absorbance of the plates was measured after being gently shaken using a test wavelength of 490 nm. All results were corrected for background absorbance detected in wells without added stain. Treated samples were compared with the cell control in the absence of the tested compounds. All experiments were carried out in triplicate. The cell cytotoxic effect of each tested compound was calculated. The optical density was measured to determine the number of viable cells, and the percentage of viability was calculated as $[(OD_t/OD_c)] \times 100$ %. The relation between surviving cells and drug concentration is plotted, and the IC_{50} was estimated [31-33].

1.3. Antioxidant Activity

1.3.1. FRAP scavenging activity

The reduction of ferric to ferrous ions by the drugs is an indication of its potential antioxidant properties. The reducing power of the isolated compounds was evaluated using the reported method [33, 34]. This method is based on the reduction of ferricyanide relative to different concentrations of the drug sample. Samples in 1mL of methanol were mixed with 2.5 mL of 0.2 M sodium phosphate buffer (pH 6.6) and 2.5 mL of potassium ferricyanide [$K_3Fe(CN)_6$] (1%, w/v). After 20 min of incubation at 50 °C, the reaction mixture was acidified with 2.5 mL of trichloroacetic acid (10%, w/v). The reaction mixture was centrifuged at 1000*xg* for 10 min. The supernatant solution (2.5 mL) was mixed with 2.5 mL of deionized water and 0.5 mL of freshly prepared ferric chloride (0.1%, w/v). The absorbance of the resulting solution was measured at 700 nm versus a blank. Ascorbic acid was used as a reference standard. The reducing capability percentage (%) was calculated as follows [31-33]**:**

$$\text{Reducing capability (\%)} = 100 - \left[\frac{Ao - As}{Ao} x100\right]$$

Where, A_o: absorbance of the control solution. As: sample absorbance.

1.3.2. ABTS Radical Scavenging Assay

The ABTS scavenging capacity method is a decolorization assay that measures the capacity of antioxidants to directly react with ABTS radicals generated by a chemical method [36]. The

determination of antioxidant activity by the ABTS radical scavenging method was performed according to the procedure described in the literature [37]. ABTS radical cation ($ABTS^{+}$) was produced by reacting ABTS stock solution (1.8 mM) with 0.63 mM potassium persulfate and allowing the mixture to stand in the dark at room temperature for 12-16 h before use. Then, the solution was diluted with ethanol until absorbance reached 0.700 (±0.030) at 734 nm. The samples were diluted at a ratio of 1:10 with methanol (80%). Later, 190µl of radical solution was mixed with 10µl of diluted drugs in a microtiter plate. The absorbance at 734 nm was measured every 1 min until 13 min following initial mixing. Appropriate solvent blanks were run in each assay. Ascorbic acid and methanol were used as the standard antioxidant and the negative control, respectively. Experiments were performed three times with three replicates for each sample. The percent free radical scavenging activity was calculated according to the following formula [31-33]:

$$\text{\% Free Radical Scavenging Activity} = \frac{A_n - A_s}{A_n} x100$$

where A_n is the final absorbance values of negative control, and A_s is the final absorbance values of sample.

1.3.3. DPPH scavenging Activity

A freshly prepared (0.004% w/v) methanol solution of 2,2-diphenyl-1-picrylhydrazyl (DPPH) radical was prepared and stored at 10 °C in the dark. A methanol solution of the test compound was prepared. A 40-µL aliquot of the methanol solution was added to 3 ml of DPPH solution. Absorbance measurements were recorded immediately with a UV-visible spectrophotometer. The decrease in absorbance at 515 nm was determined continuously, with data being recorded at 1 min intervals until the absorbance stabilized (16 min). The absorbance of the DPPH radical without an antioxidant (control) and the reference compound ascorbic acid were also measured. All the determinations were performed in three replicates and averaged. The percentage inhibition (PI) of the DPPH radical was calculated [31-33]:

$$\text{PI} = 100 - \left[\frac{Ac - At}{Ac} x100\right]$$

Where A_c = absorbance of the control at t = 0 min and A_t = absorbance of the sample + DPPH at t = 16 min. The 50% inhibitory concentration (IC_{50}), the concentration required for 50% DPPH radical scavenging activity, was estimated from graphic plots of the dose response curve.

A

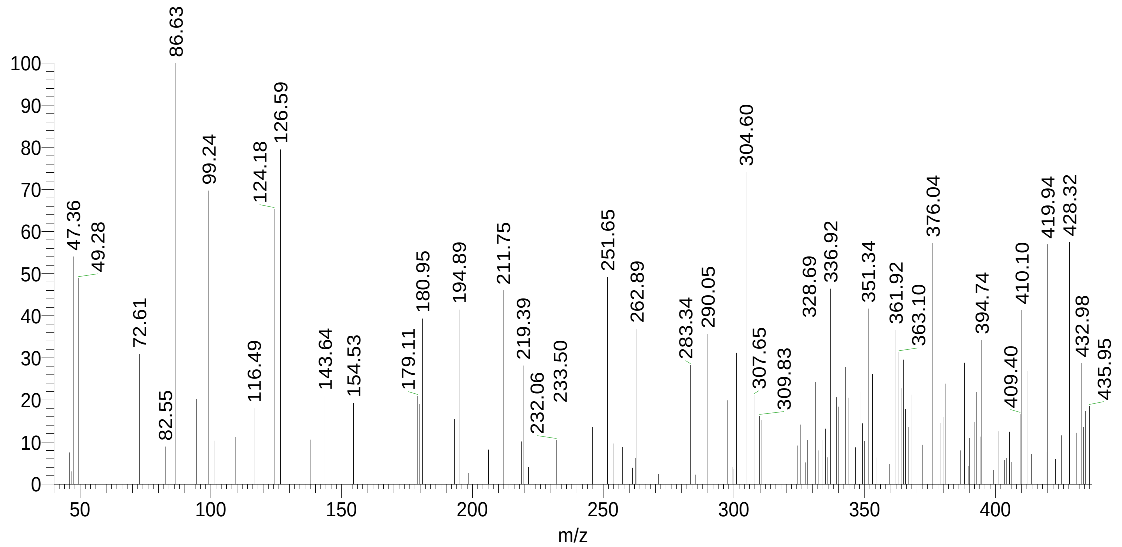

B

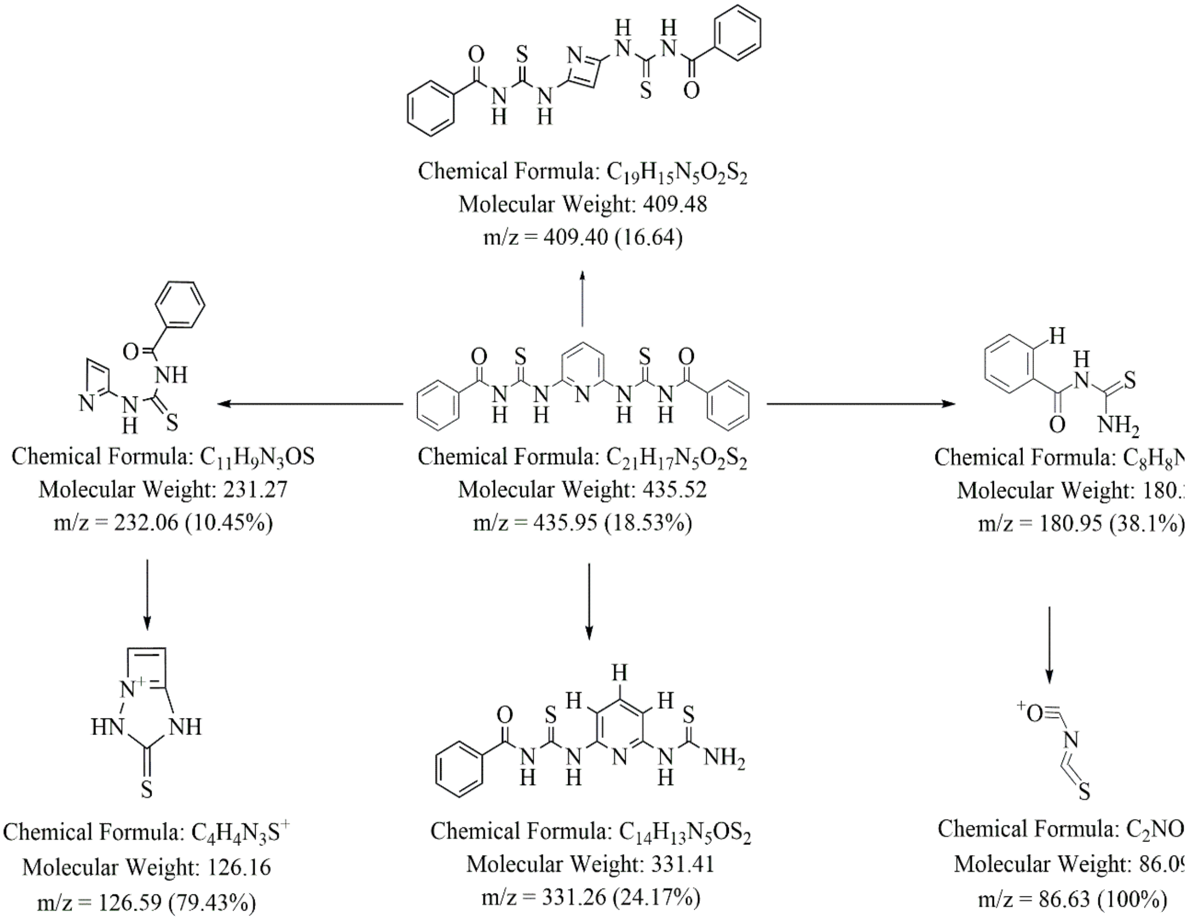

Figure S1: (A) mass spectrum of H4DAP ligand, and (B) Suggested fragmentation pattern of H_4DAP ligand.

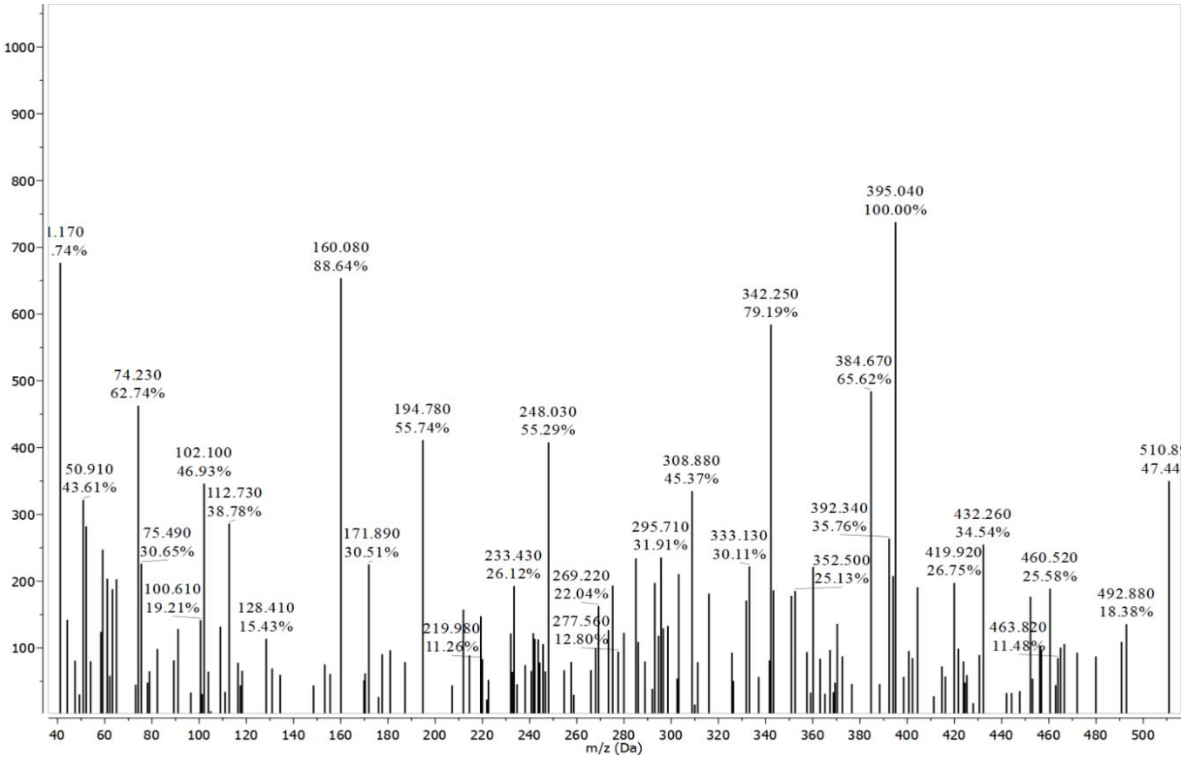

Figure S2: mass spectrum of Co^{2+} complex

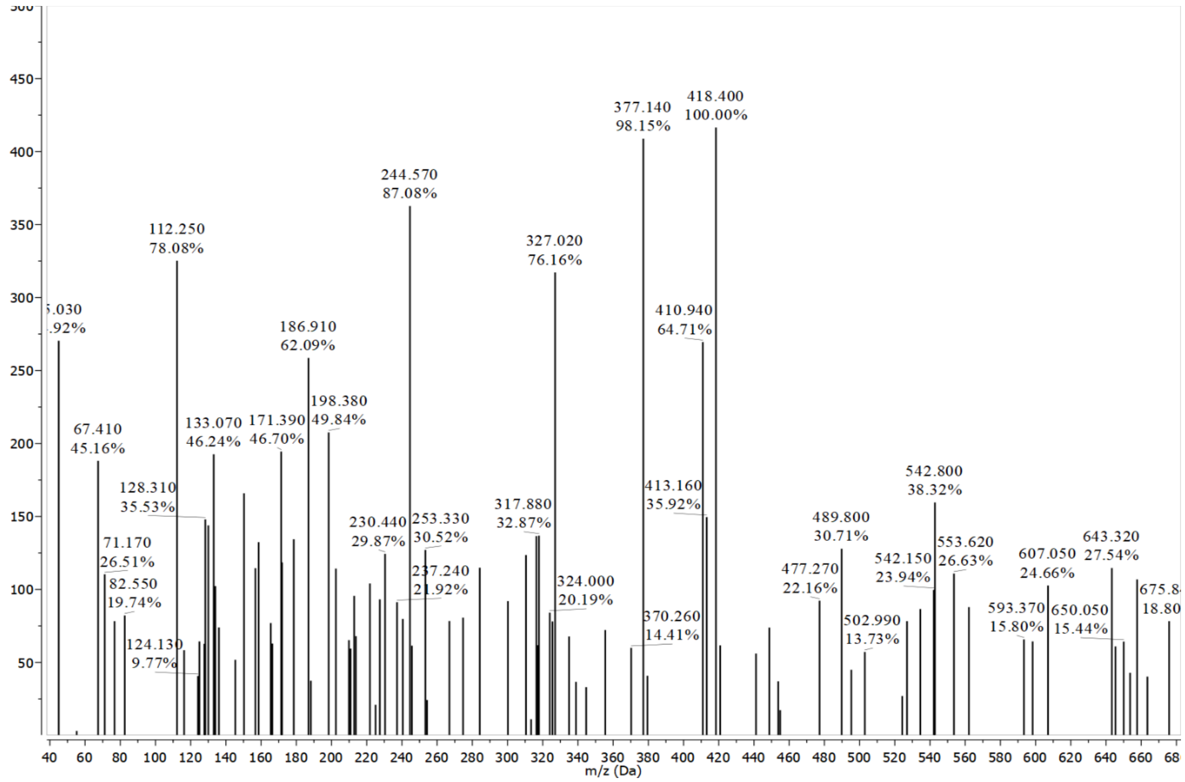

Figure S3: mass spectrum of Ni^{2+} complex

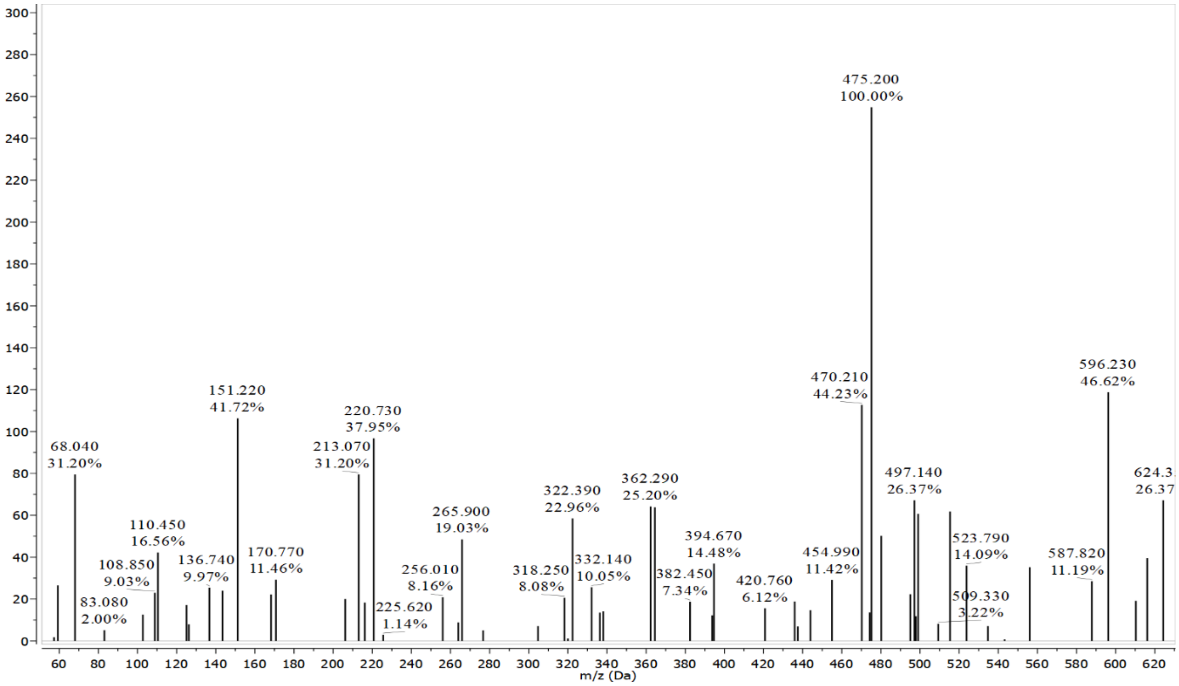

Figure S4: mass spectrum of Cu^{2+} complex

A

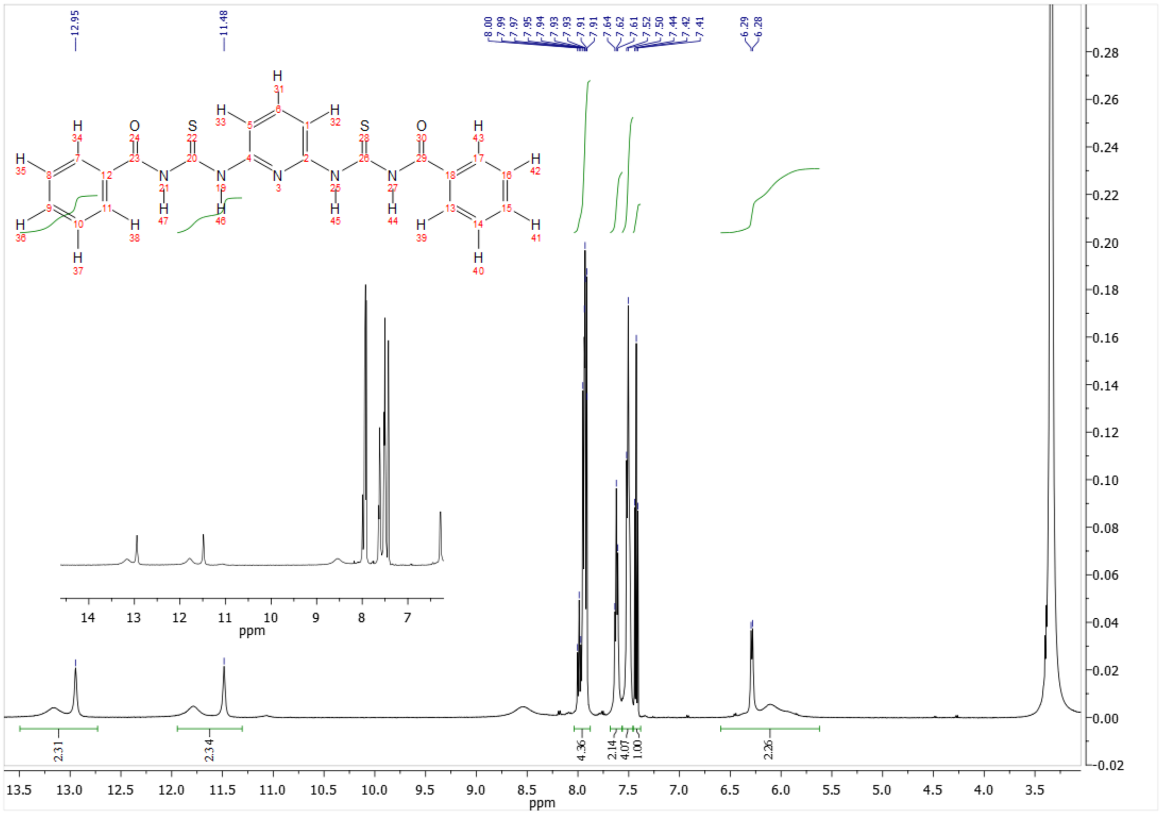

B

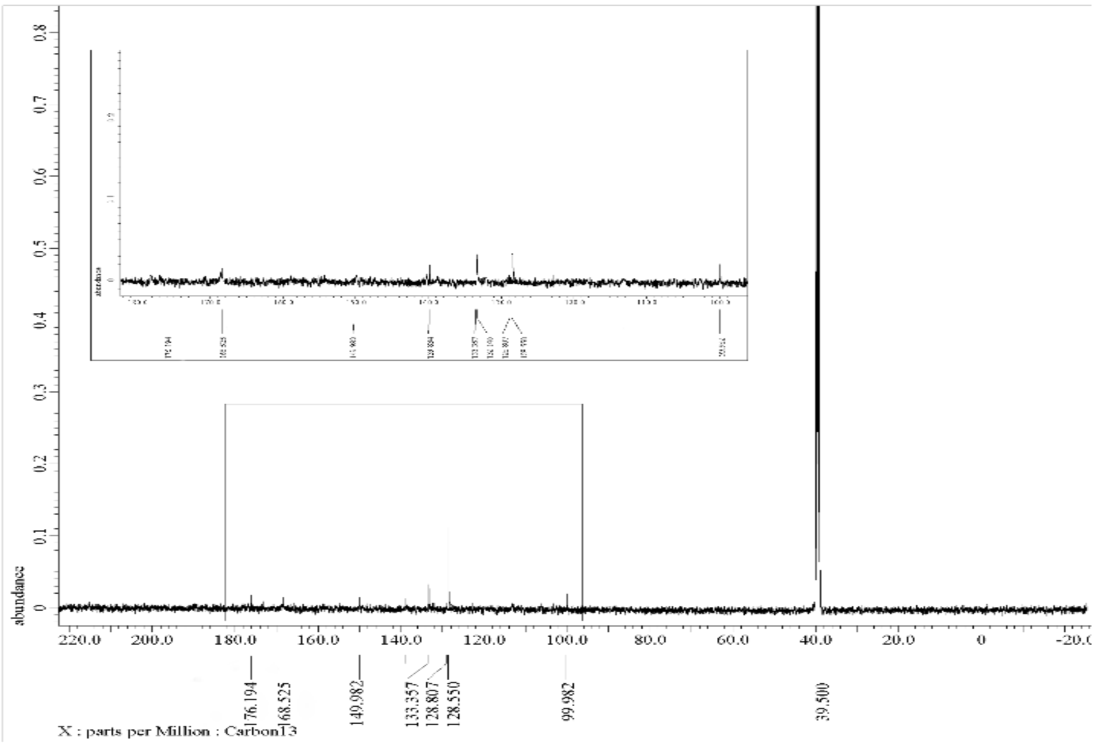

Figure S5: (A) ^{1}HNMR spectrum and (B) ^{13}CNMR spectrum of H_4DAP ligand

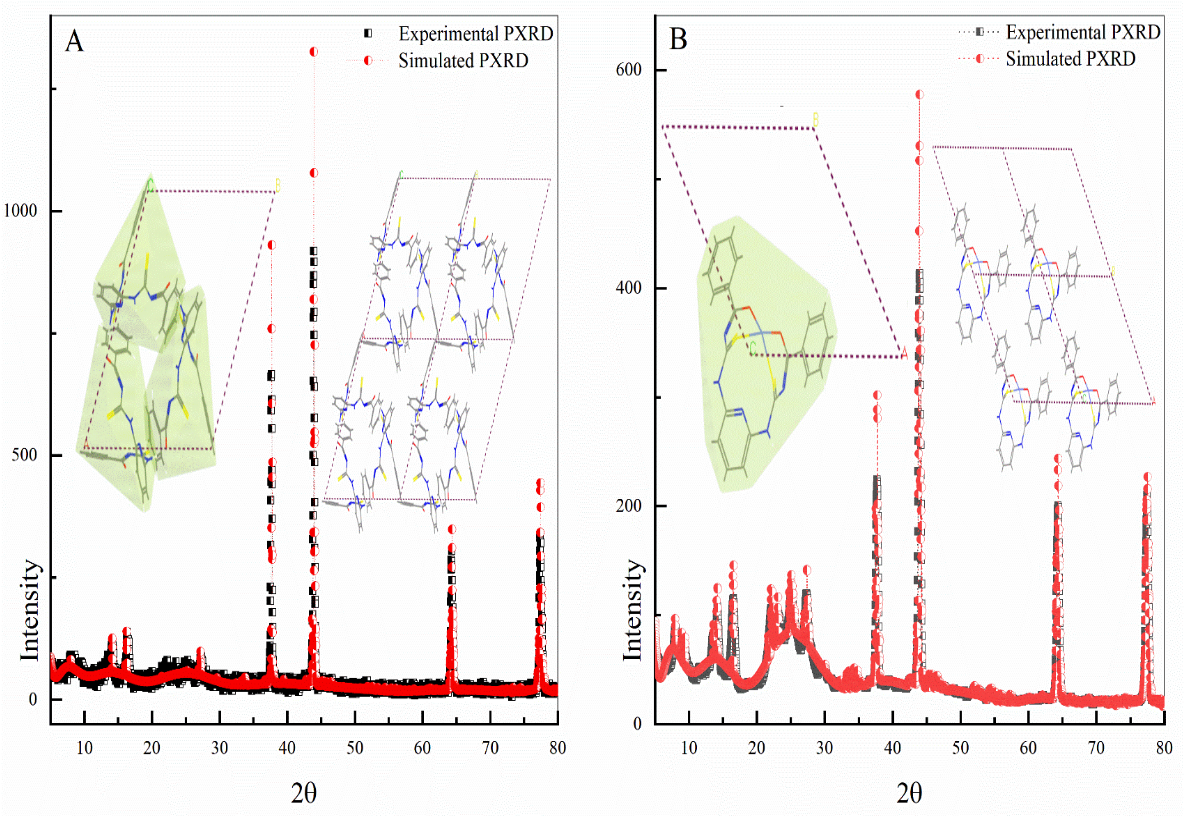

Figure S6: Experimental and simulated PXRD patterns along with simulated crystal structure for (A) H_4DAP ligand and (B) Co^{2+} complex obtained from MS.

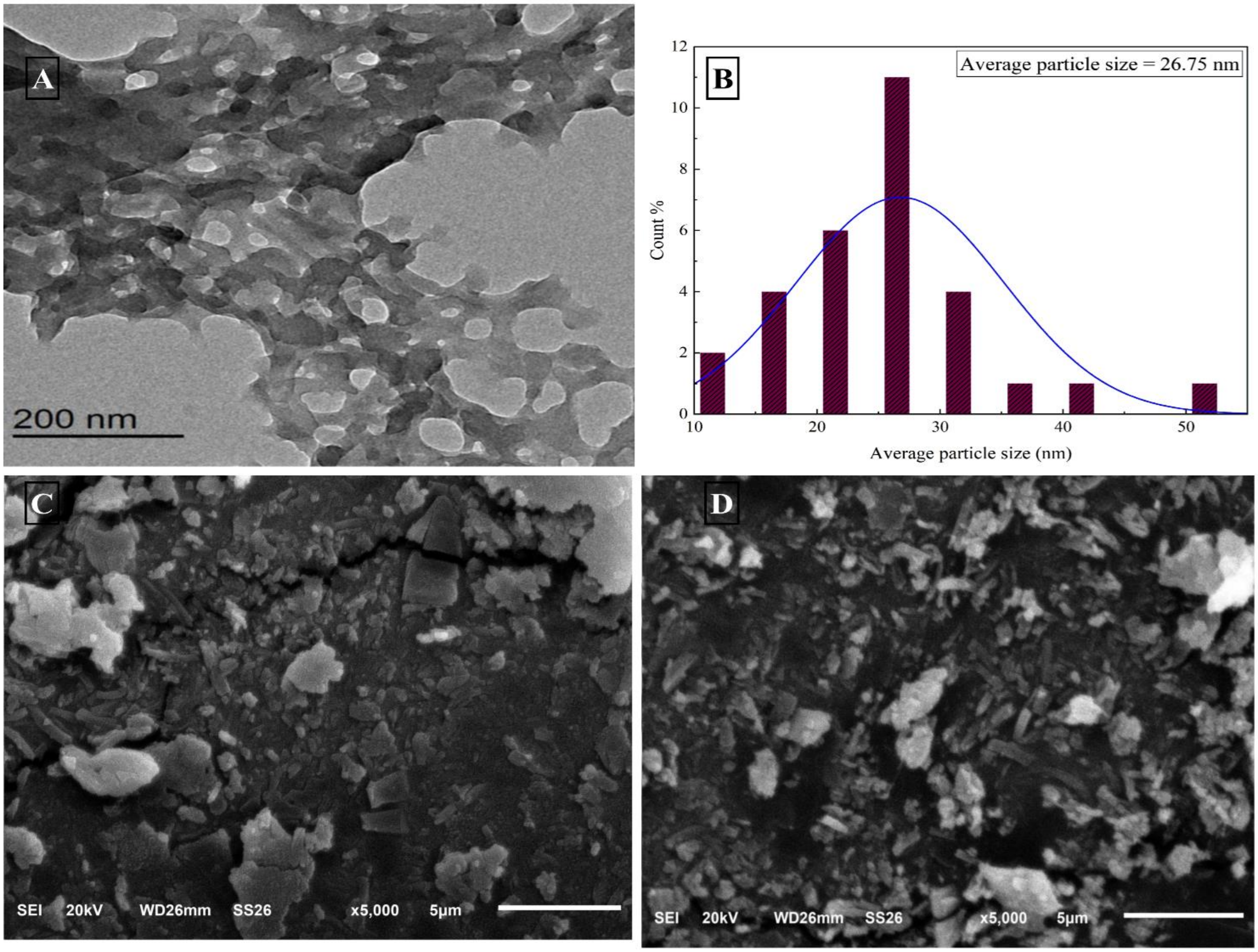

Figure S7: (A) TEM, (B) Histogram plot, (C) and (D) SEM images of H_4DAP ligand.

A

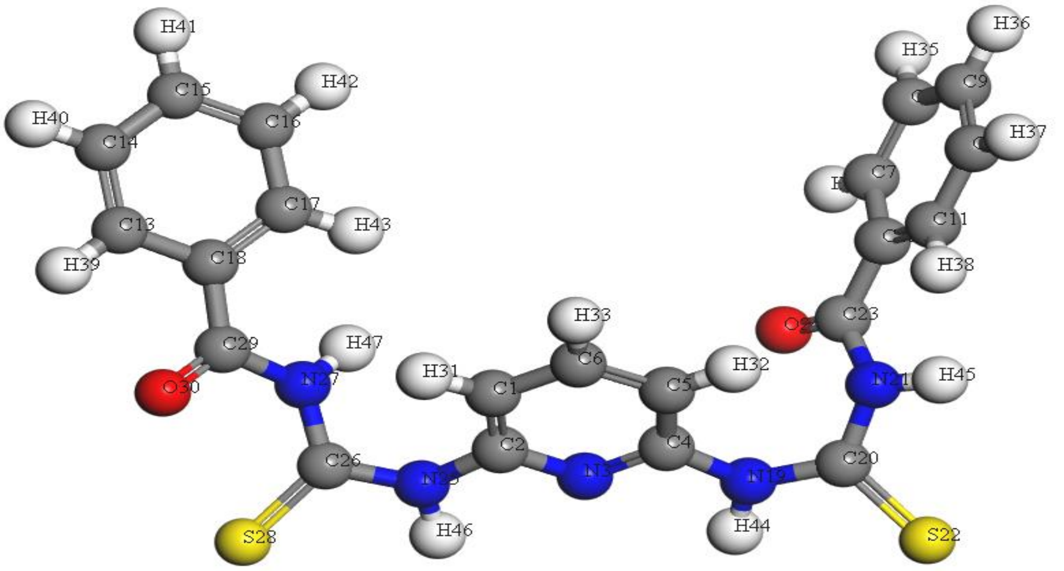
H41
C15
H42
H40
C14
C16
C17
H43
C13
H39
C18
C29
H47
O30
H31
C1
H33
C6
C5
H32
C2
C4
C26
C20
H46
H44
S28
S22
H35
H36
C9
H37
C7
C11
H38
C23
H45

B

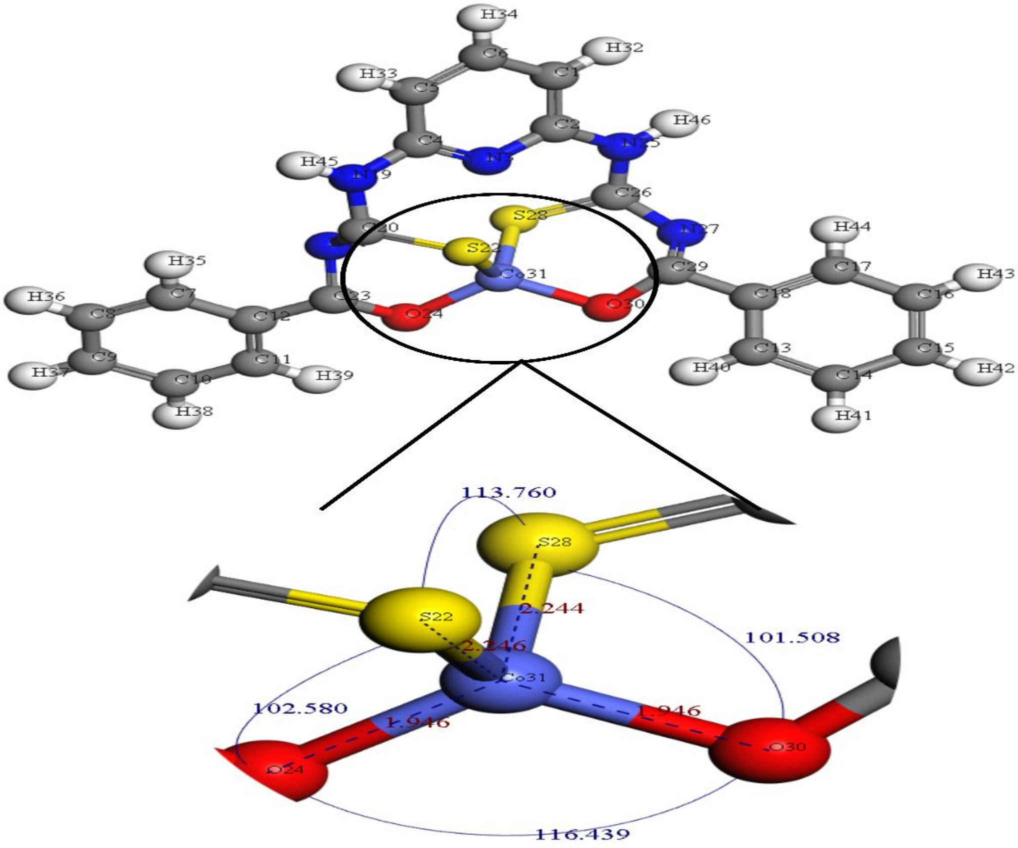
H34
H32
H33
H46
H45
S28
S22
Co31
O24
O30
C26
C29
C23
H35
H36
C7
C12
H37
C11
H39
H38
H44
C17
H43
C18
C16
H40
C13
C14
C15
H42
H41
113.760
101.508
102.580
116.439
2.244
2.246
1.946

C

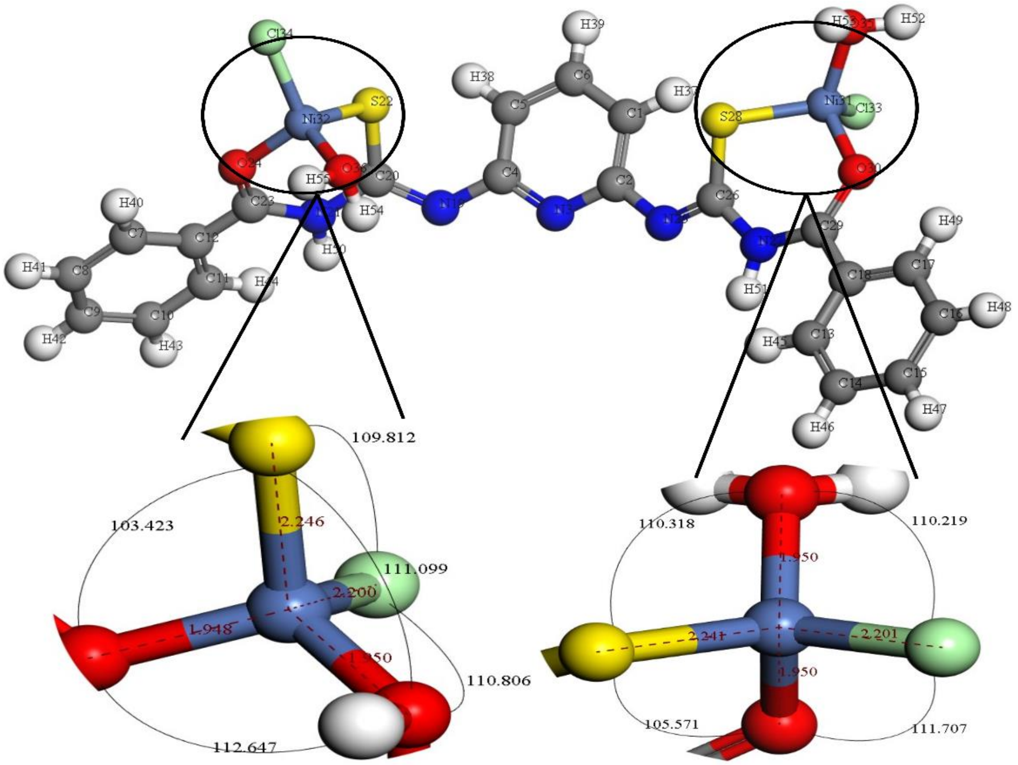

Cl34
Ni32
S22
C20
C24
C23
H55
H54
H50
H40
C7
C12
C11
H41
C8
C9
C10
H42
H43
H44
H38
C5
H39
C6
C1
H37
C4
C2
C26
S28
Ni31
Cl33
H53
H52
C29
H49
C18
C17
C16
H48
H51
H45
C13
C14
C15
H47
H46
109.812
103.423
2.246
111.099
2.200
1.948
110.806
112.647
110.318
110.219
2.241
2.201
1.950
105.571
111.707

D

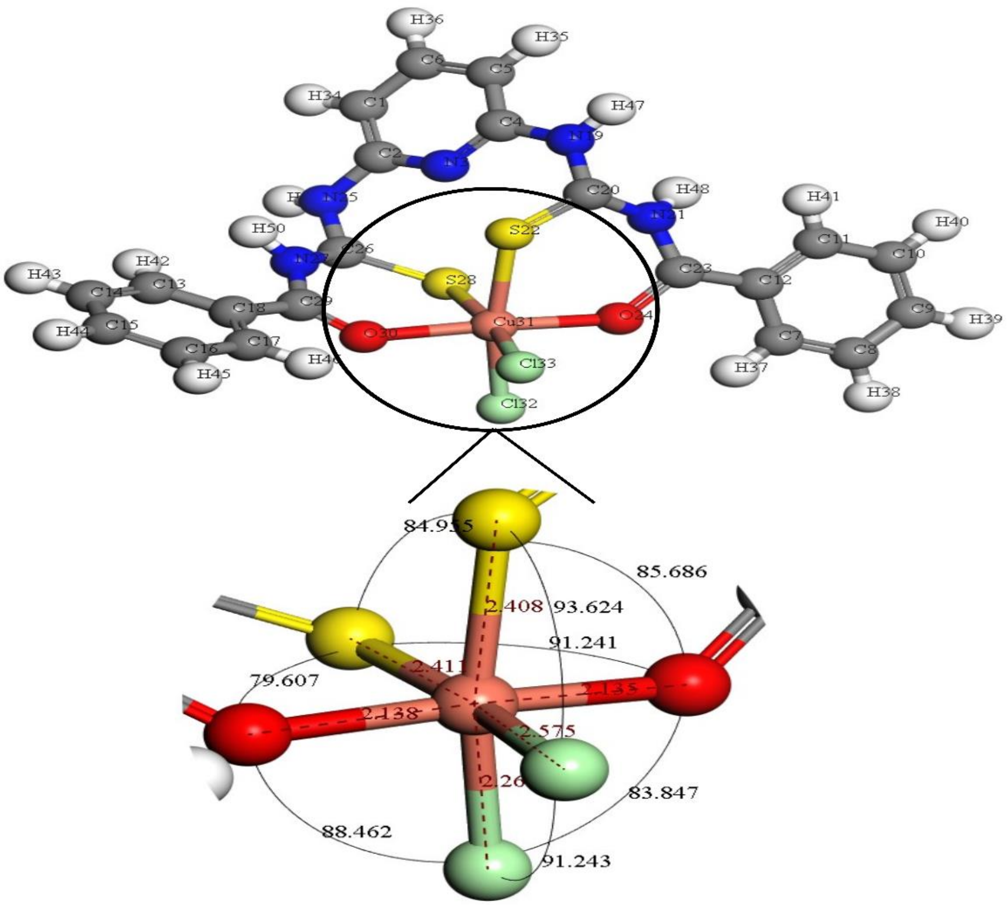

Figure S8: optimized structures of (A): H_4DAP ligand, (B): Co^{2+} complex, (C): Ni^{2+} complex and (D): Cu^{2+} complex.

A

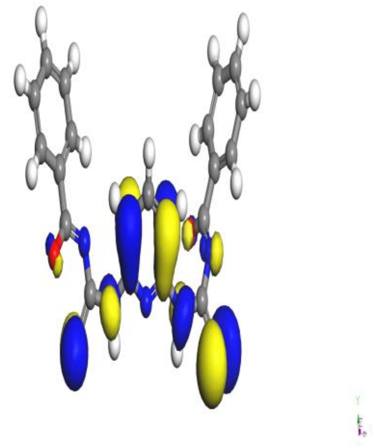

E_{HOMO} = -5.6727 eV

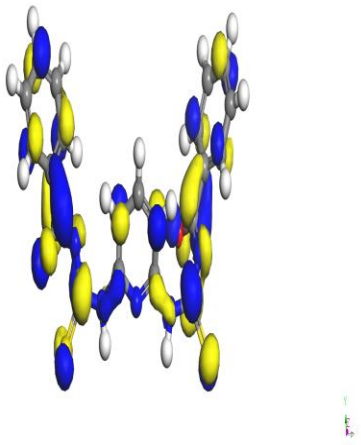

E_{LUMO} = -3.03721 eV

B

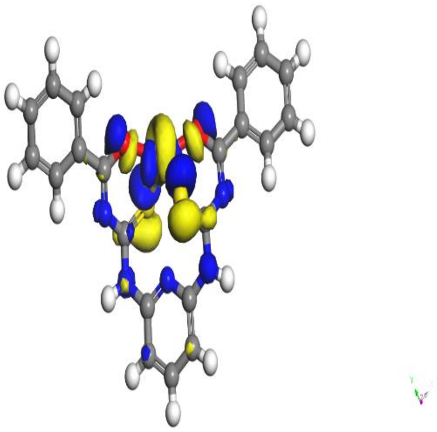

E_{HOMO} = -4.3515 eV

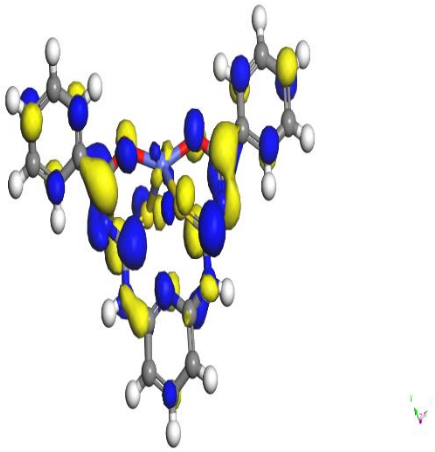

E_{LUMO} = -3.30644 eV

C

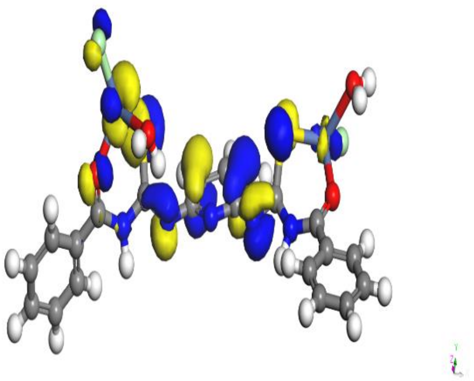

E_{HOMO} = -5.3217 eV

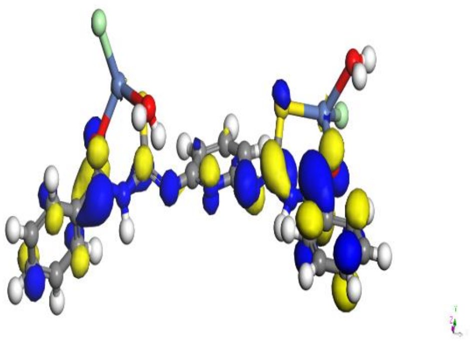

E_{LUMO} = -3.31158 eV

D

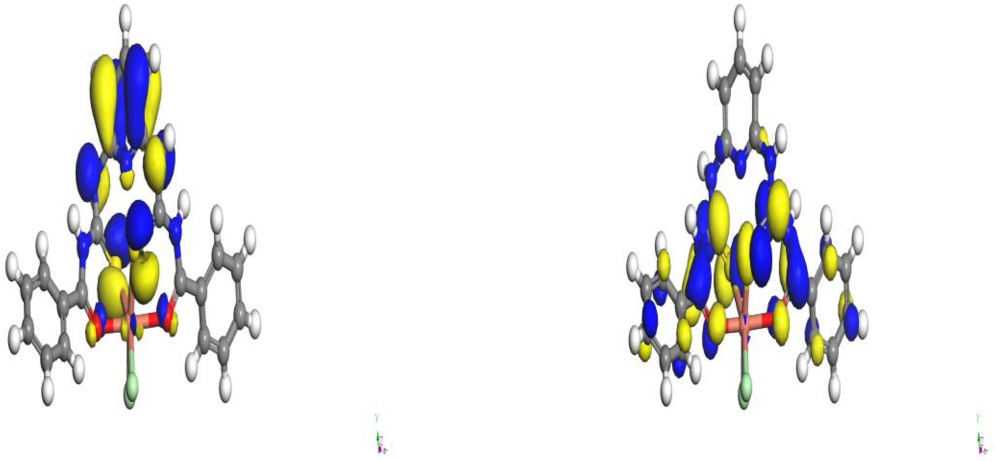

E $_{HOMO}$ = -5.5566 eV   E $_{LUMO}$ = -3.6208 eV

Figure S9: HOMO and LUMO orbitals of (A): H_4DAP ligand, (B): Co^{2+} complex, (C): Ni^{2+} complex and (D): Cu^{2+} complex.

A

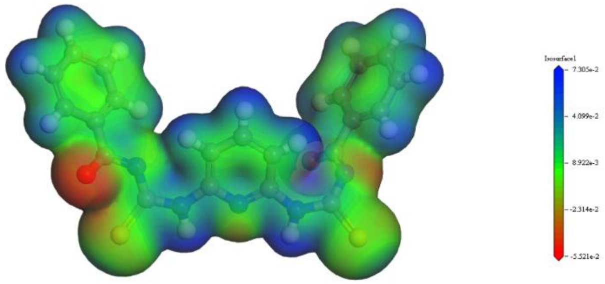

B

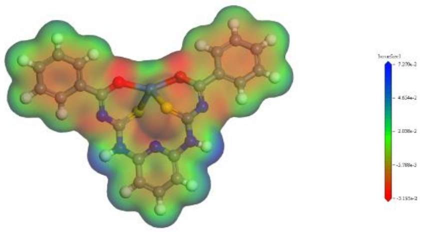

C

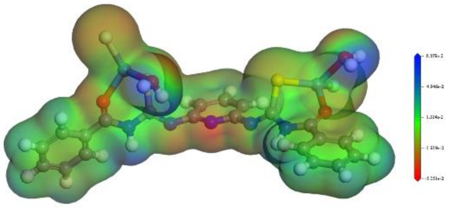

D

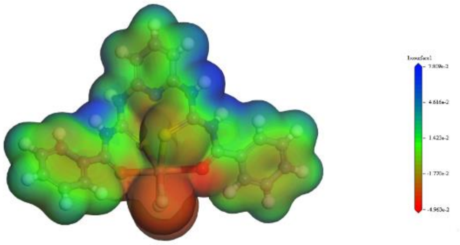

Figure S10: MEP maps of (A): H_4DAP ligand, (B): Co^{2+} complex, (C): Ni^{2+} complex and (D): Cu^{2+} complex.

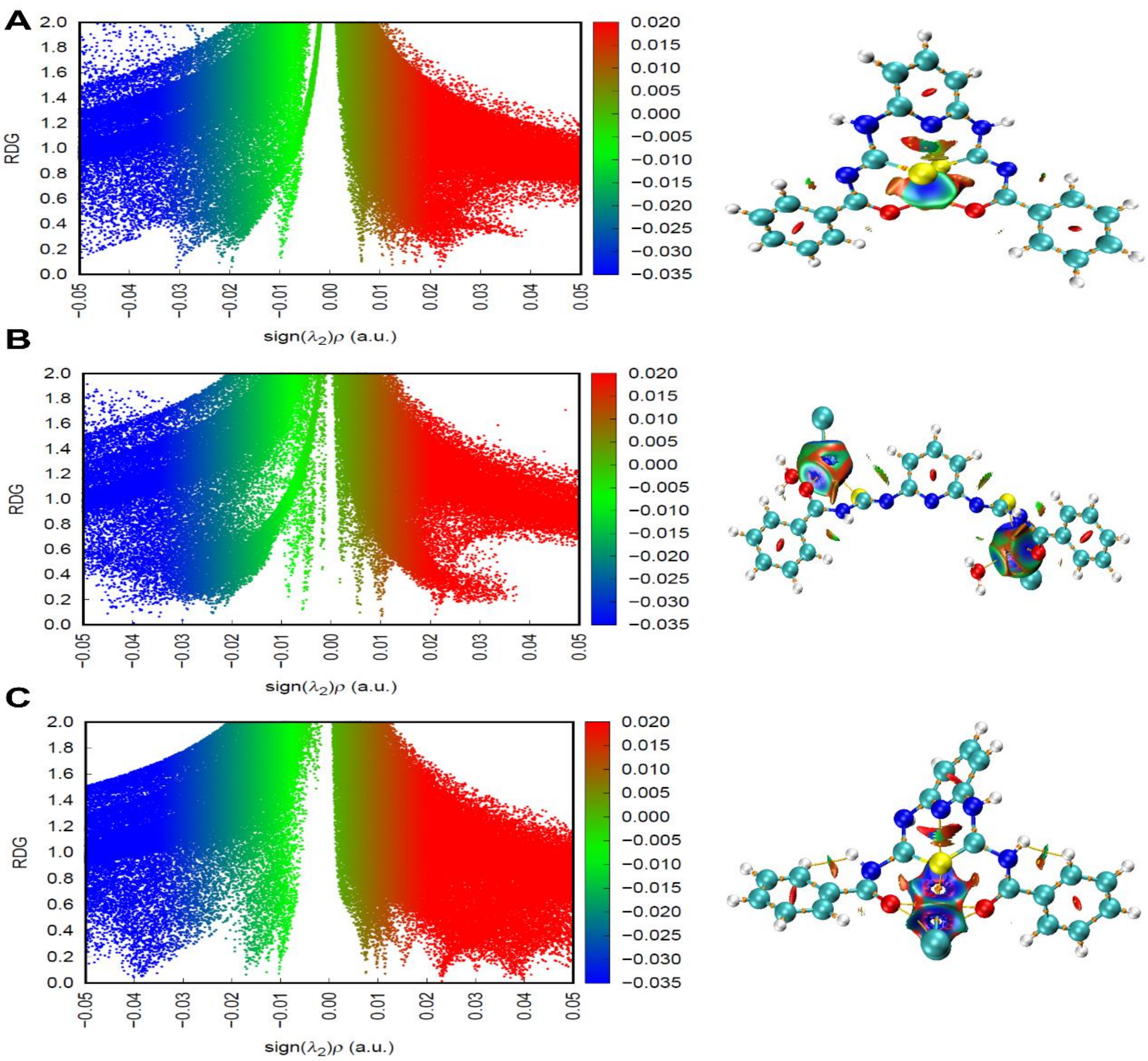

Figure S11: AIM molecular graphs of (A) Co^{2+}, (B) Ni^{2+} and (C) Cu^{2+} complexes.

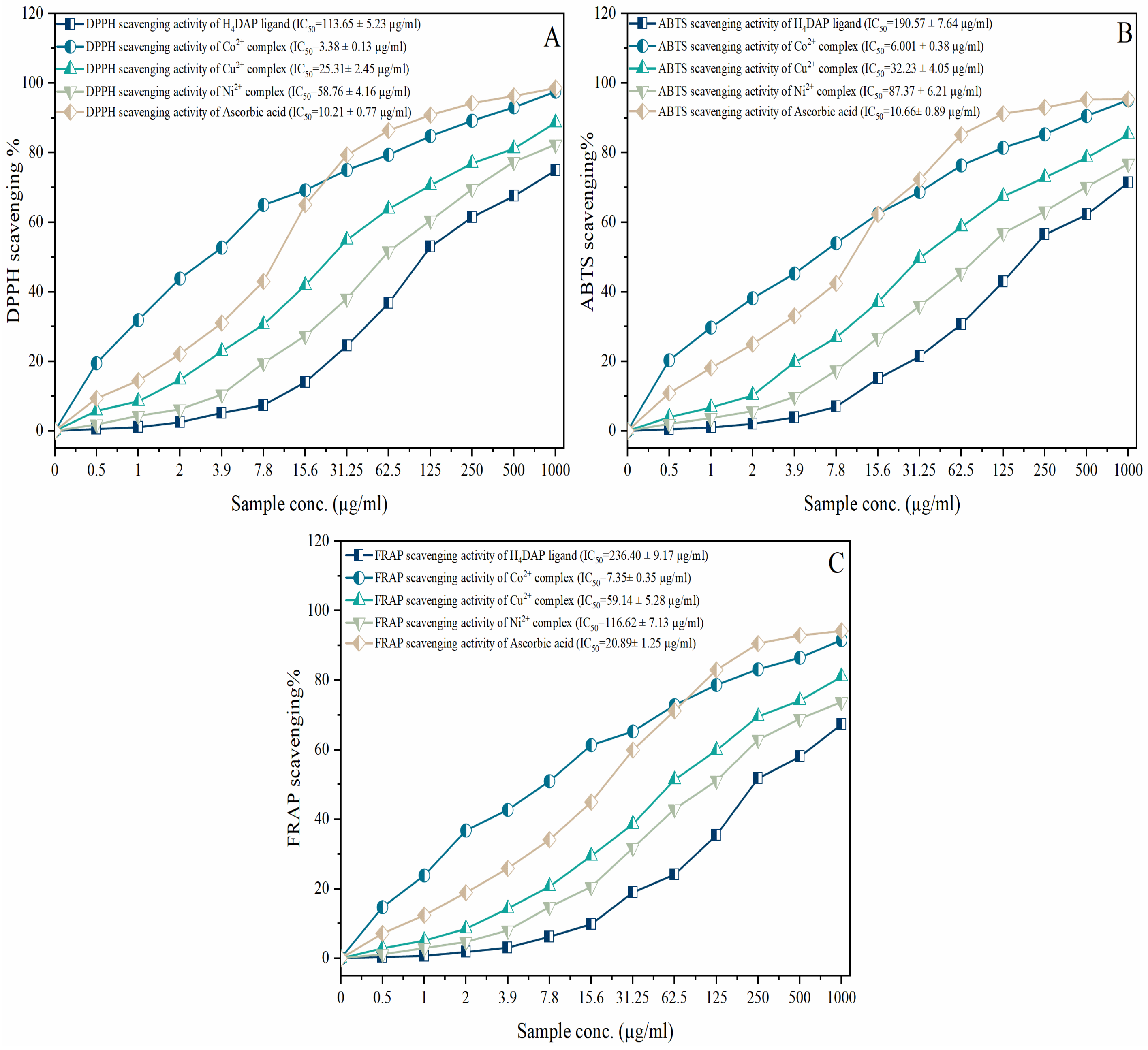

Figure S12: Antioxidant Activity of H_4DAP ligand and its complexes using: (A) DPPH, (B) ABTS and (D) FRAP scavenging assays.

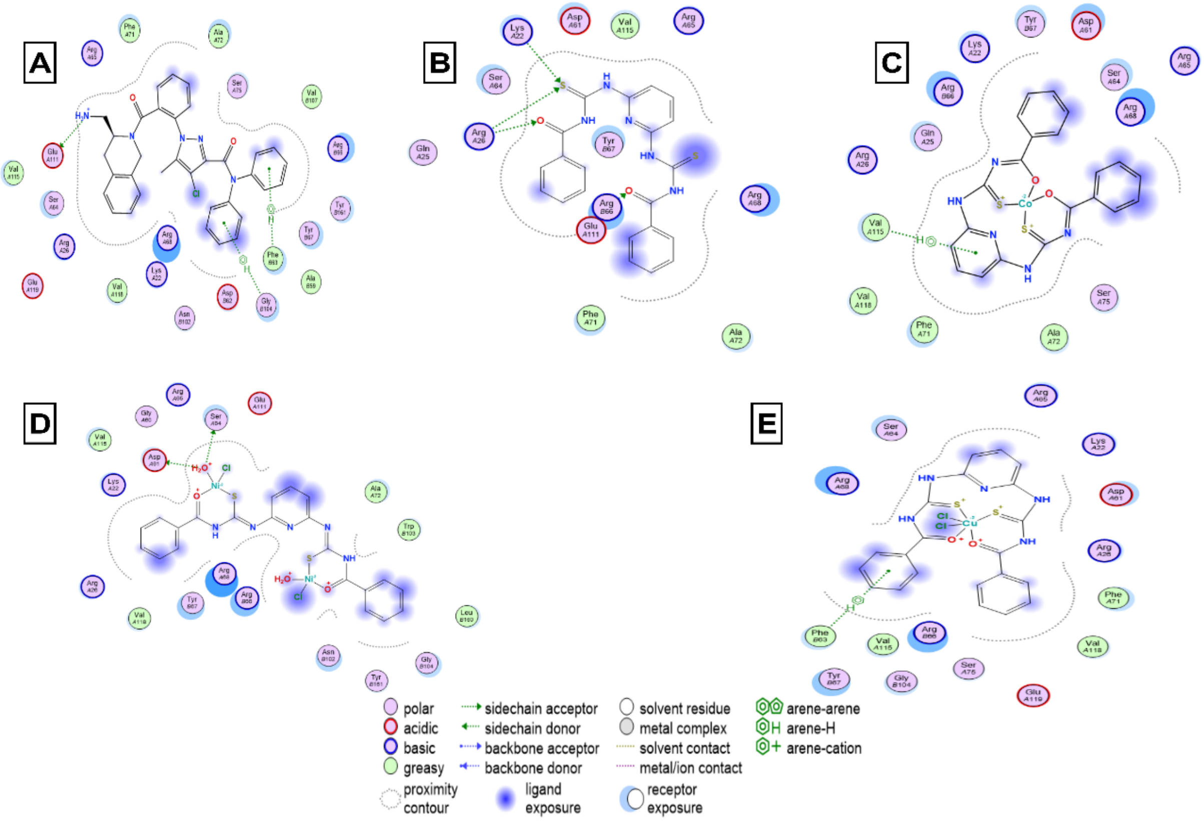

Figure S13: 2D docking possess of: A) DRO reference ligand, B) H_4DAP ligand, C) Co^{2+} complex, D) Ni^{2+} complex, and E) Cu^{2+} complex

Table S1: XRD data of H_4DAP ligand and its complexes; symmetry, space group, cell parameters, FWHM (β), d-Spacing, Grain size (D), Micro strain (ε) and dislocation density (δ)

Compound/Crystal system (Space group) and cell parameters	2Θ°	(β)	d (Å)	D	ε	δ
**H_4DAP Ligand**<br>Hexagonal *(R3C)*<br>a = 14.466 Å<br>b = 14.466 Å<br>c = 11.035 Å<br>α = 90 °<br>β = 90 °<br>γ = 120 °	8.025	0.41	11.00836	19.42393	0.00265	0.025503
	14.137	0.374	6.259757	21.40409	0.002183	0.013161
	16.432	0.536	5.390282	14.97513	0.004459	0.016198
	27.2566	0.3667	3.269217	22.29191	0.002012	0.006599
	37.6661	0.2192	2.386221	38.29224	0.000682	0.002804
	43.9124	0.2176	2.060189	39.36373	0.000645	0.002355
	64.2842	0.238	1.447875	39.42144	0.000643	0.001653
	77.4027	0.2692	1.231965	37.81412	0.000699	0.001466
**[Co(H_2DAP)].H_2O**<br>Triclinic *(P-1)*<br>a = 7.113 Å<br>b = 12.378 Å<br>c = 13.835 Å<br>α = 62.72 °<br>β = 113.54 °<br>γ = 75.98 °	8.02	0.36	11.01521	22.12163	0.002043	0.022407
	8.68	0.4	10.17907	19.91784	0.002521	0.022997
	9.5	0.256	9.302218	31.13933	0.001031	0.013443
	13.565	0.33	6.522401	24.24327	0.001701	0.012107
	14.208	0.344	6.228635	23.27252	0.001846	0.012044
	16.5575	0.565	5.349707	14.20875	0.004953	0.016943
	22.04	0.48	4.029785	16.86152	0.003517	0.010755
	22.44	0.32	3.958849	25.30964	0.001561	0.007039
	22.8	0.2	3.897148	40.52087	0.000609	0.004328
	23.04	0.27	3.857093	30.02821	0.001109	0.00578
	23.24	0.24	3.82435	33.79381	0.000876	0.005093
	23.46	0.24	3.788982	33.80722	0.000875	0.005043
	24.8	0.2266	3.587207	35.89602	0.000776	0.004497
	25.3	0.32	3.517435	25.44352	0.001545	0.006221
	25.58	0.2666	3.479564	30.55671	0.001071	0.005124
	27.04	0.14	3.294911	58.36223	0.000294	0.002541
	27.3	0.44	3.264118	18.57999	0.002897	0.007906
	27.58	0.36	3.231613	22.72242	0.001937	0.0064
	37.6981	0.2211	2.384269	37.9668	0.000694	0.002826
	43.64	0.1	2.072415	85.57372	0.000137	0.00109

	43.9472	0.2469	2.058639	34.69663	0.000831	0.00267
	64.3158	0.2451	1.44724	38.28613	0.000682	0.001701
	77.4156	0.2731	1.231792	37.27748	0.00072	0.001487
	77.78	0.12	1.22693	85.05451	0.000138	0.000649
**$[Ni_2(H_2DAP)Cl_2(H_2O)_2].H_2O$** Monoclinic (*C2/C*), a = 13.55Å, b = 4.47Å, c = 7.70Å, α = 90°, β = 118.89°, γ = 90°	26.8823	0.3865	3.313881	21.13329	0.002239	0.007056
	23.0187	0.3575	3.860614	22.67779	0.001944	0.007661
	13.24	0.48	6.681766	16.66169	0.003602	0.018046
**$[Cu(H_4DAP)Cl_2].3H_2O$** Triclinic (P-1), a = 5.81Å, b = 13.08Å, c = 13.95Å, α = 44.41°, β = 95.50°, γ = 92.84°	43.899	0.202	2.060787	42.40171	0.000556	0.002187
	77.4223	0.2619	1.231703	38.87345	0.000662	0.001426
	37.6683	0.2233	2.386087	37.5894	0.000708	0.002856

Table S2: Calculated E_H, E_L, energy band gap (E_H-E_L), chemical potential (μ), electronegativity (χ), global hardness (η), global softness (S) and global electrophilicity index (ω) for H_3L ligand and its metal complexes

compound	E_H/eV	E_L/eV	(E_H-E_L) /eV	χ /eV	μ /eV	η/eV	S/eV^{-1}	ω/eV
H_4DAP Ligand	-5.6727	-3.03721	-2.63555	-4.354984495	4.354984	1.317774308	0.658887	7.196
$[Co(H_2DAP)].H_2O$	-4.3515	-3.30644	-1.04512	-3.828995865	3.828996	0.522560044	0.26128	14.028
$[Ni_2(H_2DAP)Cl_2(H_2O)_2].H_2O$	-5.3217	-3.31158	-2.01013	-4.316644196	4.316644	1.005065496	0.502533	9.269
$[Cu(H_4DAP)Cl_2].3H_2O$	-5.5566	-3.6208	-1.93584	-4.588726985	4.588727	0.967922481	0.483961	10.877

Table S3: topological parameters for selected bonds of interacting atoms, Laplacian of electron density ($\nabla^2\rho_b$), electron kinetic energy density (G_b), electron potential energy density (V_b), total energy density (H) at BCP (3, -1).

Ligand						
BCP #, (Atoms)	$\nabla^2\rho_b$	V_b	G_b	H_b	G_b/V_b	E_n
66 (H^{31} - N^{27})	0.048	-0.008	0.010	0.004	-1.225	-0.004
72 (H^{32} - N^{21})	0.037	-0.006	0.008	0.002	-1.317	-0.003
79 (C^{4} - O^{24})	0.041	-0.008	0.009	0.001	-1.113	-0.004
74 (N^{19} - O^{24})	0.044	-0.008	0.009	0.002	-1.170	-0.004
83 (H^{45} - H^{38})	0.054	-0.008	0.011	0.003	-1.359	-0.004

Co complex						
BCP #, (Atoms)	$\nabla^2\rho_b$	V_b	G_b	H_b	G_b/V_b	E_n
68 (O^{30} - Co^{31})	1.446	-0.252	0.306	0.055	-1.216	-0.126
70 (O^{24} - Co^{31})	1.513	-0.267	0.323	0.055	-1.209	-0.134
78 (Co^{31} - S^{28})	0.151	-0.034	0.036	0.002	-1.053	-0.017
79 (Co^{31} - S^{22})	0.342	-0.076	0.081	0.005	-1.062	-0.038
88 (S^{28} - N^{3})	0.106	-0.017	0.022	0.005	-1.273	-0.009

Ni complex						
BCP #, (Atoms)	$\nabla^2\rho_b$	V_b	G_b	H_b	G_b/V_b	E_n
58 (O^{35} - Ni^{31})	0.102	-0.026	0.026	0.000	-0.993	-0.013
59 (Cl^{33} - Ni^{31})	0.340	-0.069	0.077	0.008	-1.119	-0.035
60 (Ni^{31} - S^{28})	0.303	-0.067	0.071	0.004	-1.062	-0.034
60 (Ni^{31} - O^{30})	0.321	-0.056	0.068	0.012	-1.219	-0.028
63 (Cl^{34} - Ni^{32})	0.375	-0.076	0.085	0.009	-1.113	-0.038
68 (S^{22} - Ni^{32})	0.309	-0.068	0.073	0.005	-1.067	-0.034
73 (Ni^{32} - O^{36})	0.077	-0.019	0.020	0.000	-1.029	-0.009
76 (Ni^{32} – O^{24})	0.295	-0.052	0.063	0.011	-1.207	-0.026

Cu complex						
BCP #, (Atoms)	$\nabla^2\rho_b$	V_b	G_b	H_b	G_b/V_b	E_n
52 (Cl^{33} – Cl^{32})	0.072	-0.024	0.021	-0.003	-0.881	-0.012
56 (Cl^{33} – Cu^{31})	0.525	-0.099	0.115	0.016	-1.165	-0.049
57 (Cl^{33} – O^{30})	0.088	-0.024	0.023	-0.001	-0.963	-0.012
61 (Cl^{32} – Cu^{31})	0.523	-0.099	0.115	0.016	-1.163	-0.049
62 (Cl^{32} – O^{24})	0.087	-0.024	0.023	-0.001	-0.957	-0.012
67 (O^{30} – Cu^{31})	0.941	-0.148	0.192	0.044	-1.294	-0.074
68 (O^{24} – Cu^{31})	0.932	-0.147	0.190	0.043	-1.292	-0.074
73 (Cu^{31} – S^{22})	0.503	-0.103	0.114	0.114	-1.110	-0.052
82 (Cu^{31} – S^{28})	0.515	-0.102	0.115	0.014	-1.129	-0.051
89 (S^{22} – S^{28})	0.064	-0.027	0.022	-0.006	-0.805	-0.014
